# Evaluation of bisulfite kits for DNA methylation profiling in terms of DNA fragmentation and DNA recovery using digital PCR

**DOI:** 10.1371/journal.pone.0199091

**Published:** 2018-06-14

**Authors:** Sam Kint, Ward De Spiegelaere, Jonas De Kesel, Linos Vandekerckhove, Wim Van Criekinge

**Affiliations:** 1 Department of Data Analysis and Mathematical Modelling, Faculty of Bioscience Engineering, Ghent University, Ghent, Belgium; 2 HIV Cure Research Center, Department of Internal Medicine, Faculty of Medicine and Health Sciences, Ghent University and Ghent University Hospital, Ghent, Belgium; 3 Department of Morphology, Faculty of Veterinary Medicine, Ghent University, Merelbeke, Belgium; 4 Department of Biotechnology, Faculty of Bioscience Engineering, Ghent University, Ghent, Belgium; University of Perugia, ITALY

## Abstract

DNA methylation is one of the most important epigenetic modifications in the regulation of gene transcription. The current gold standard to study this modification is bisulfite sequencing. Although multiple commercial bisulfite treatment kits provide good conversion efficiencies, DNA loss and especially DNA fragmentation remain troublesome. This hampers DNA methylation profiling of long DNA sequences. Here, we explored the performance of twelve commercial bisulfite kits by an in-depth comparison of DNA fragmentation using gel electrophoresis, qPCR and digital PCR, DNA recovery by spectroscopic measurements and digital PCR and conversion efficiency by next generation sequencing. The results show a clear performance difference between the bisulfite kits, and depending on the specific goal of the study, the most appropriate kit might differ. Moreover, we demonstrated that digital PCR is a valuable method to monitor both DNA fragmentation as well as DNA recovery after bisulfite treatment.

## Introduction

DNA methylation (5-methylcytosine) is an epigenetic modification that is typically associated with stable transcriptional silencing [1–4]. This modification plays an important role in several biological processes associated with development and disease. Examples are cell differentiation, regulation of gene expression, X-chromosome inactivation and genomic imprinting [1,5,6]. In disease, DNA methylation is heavily involved in the development of genetic diseases, carcinogenesis and silencing of intracellular viruses [7–17]. Consequently, DNA methylation provides a promising diagnostic tool in medicine. Previous studies to understand the exact role of DNA methylation in these disease settings have already resulted in several clinically validated biomarkers (e.g. *MGMT* promoter methylation in patients with glioblastoma (PredictMDx, MDxHealth, Inc.); methylation of *GSTP1*, *APC* and *RASSF1* genes for prostate cancer testing (ConfirmMDx, MDxHealth, Inc.); methylation of *PITX2* in Formalin-Fixed, Paraffin-Embedded prostatectomy specimens for identifying patients who are at high risk to suffer from prostate-specific antigen recurrence after radical prostatectomy; free-circulating methylated *SEPT9* gene copies in plasma as a screening biomarker for colorectal cancer; *SHOX2* DNA methylation as a plasma based biomarker for detection of lung cancer) [18–28].

**Fig 1:**
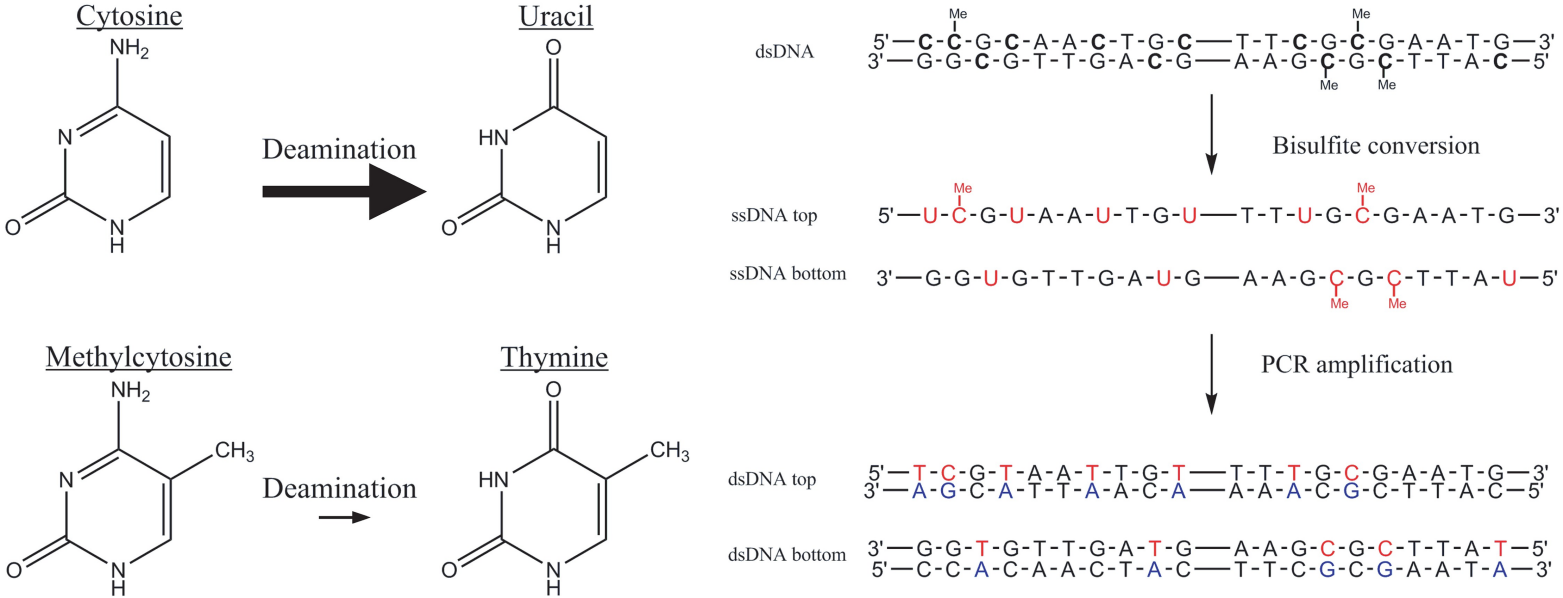
Principle of bisulfite-mediated methylcytosine (mC) mapping. A: Deamination of cytosine (C) and mC. Sodium bisulfite deaminates C to uracil (U) (upper row) and mC to thymine (T) (lower row). The rate of mC deamination is two orders of magnitude less than that of C. B: The mapping protocol: after bisulfite treatment, mC will remain C where unmethylated C will be deaminated to U. During subsequent PCR amplification, the U deamination product templates adenine (A), which then templates T, resulting in a C to T transition at unmethylated C. By sequencing, mC can be identified as bases that remained C after bisulfite treatment [38].

The analysis of DNA methylation profiles is typically done through methylation-specific PCR or bisulfite sequencing of DNA [29,30]. Bisulfite (HSO_3_^-^) treatment is first described by Frommer et al. [29] in 1992, and it is still the gold standard to analyze DNA methylation. This chemical deaminates unmethylated cytosines (C), but not methylated cytosines (mC) to uracil (U), enabling the analysis of the methylation profile through sequencing (Fig 1) [29,31]. Despite its wide use, bisulfite treatment has its disadvantages. Bisulfite conversion causes DNA fragmentation, resulting in small sequences, typically smaller than 500 nucleotides (nt) [32–35]. This is a result of the aggressive reaction condition of this conversion: pH 5 and temperatures up to 90°C [22,32,33,36]. The DNA fragmentation is mainly caused by depyrimidation followed by alkali treatment, which leads to abasic sites, resulting by DNA cleavage of the DNA phosphodiester bond (i.e. DNA degradation) [36]. Consequently, the analysis of the methylation of large CpG-islands (CpGIs) is hampered [37].

It is assumed that longer reaction times and higher conversion temperatures cause relatively more degradation [22,32,33]. However, if the temperature is too low or the reaction time is too short, the conversion might be incomplete, resulting in an overestimation of the methylation in the analyzed fragments. Therefore, the final conversion reaction has to be balanced out between desired (conversion of Cs) and undesired effects (DNA fragmentation and inappropriate conversion (conversion of mC)) [22]. A number of studies have compared the performance of different commercially available bisulfite kits, however, fragmentation or degradation of the treated DNA was never the focus of these studies [22,39–41]. Yet, this issue seems very important for some studies where the analysis of long fragments is necessary [38,42]. For example, in the HIV genome, an important CpGI is situated in the 5' Long Terminal Repeat (LTR) region, and the genome is flanked by two identical LTRs [38]. Yet, mainly the 5' LTR is of interest, since this LTR contains the promoter region [10,11,38]. To determine the methylation pattern of the promoter region of the HIV genome, we have to be able to discriminate the 5’ from the 3’ LTR. This can only be performed by amplifying DNA fragments covering the LTR as well as its a flanking region. As a result, DNA fragments have to be longer than 600 nt, which is problematic for the analysis after bisulfite treatment. The issue of DNA fragmentation has also been observed by Meissner et al. [42] in the development of an approach for a large-scale high-resolution DNA methylation analysis termed reduced representation bisulfite sequencing. In this protocol, DNA is digested, and fragments of 500–600 nt are selected, and analyzed via bisulfite sequencing.

DNA yield analysis of bisulfite kits is typically performed with spectroscopic measurements and DNA fragmentation analysis is performed by gel electrophoresis or with quantitative real-time PCR (qPCR). In these cases, multiple analysis methods are necessary. Digital PCR (dPCR) would enable us to assess the DNA yield and fragmentation with one single method. This absolute quantification method bypasses bias from PCR inhibition since it provides an end-point measurement of the positive signal enabling us to directly compare the DNA product prior to bisulfite treatment with the product after treatment. Because of the absolute quantification, it can replace multiple measurements with qubit, gel electrophoresis and qPCR.

In the current study, we provide a comprehensive procedure using state of the art technologies as dPCR to evaluate bisulfite treatment protocols by comparing twelve commercially available bisulfite kits, with focus on DNA fragmentation. The evaluation procedure consists of spectroscopic measurements and dPCR (DNA yield), gel electrophoresis, qPCR and dPCR (DNA degradation) and next generation sequencing (conversion efficiency). We show that dPCR is a valuable method to investigate the quality of bisulfite treated DNA. By using dPCR, our workflow provides a method for differentiation of DNA loss from DNA fragmentation.

## Results

### Evaluation of DNA recovery

**Fig 2:**
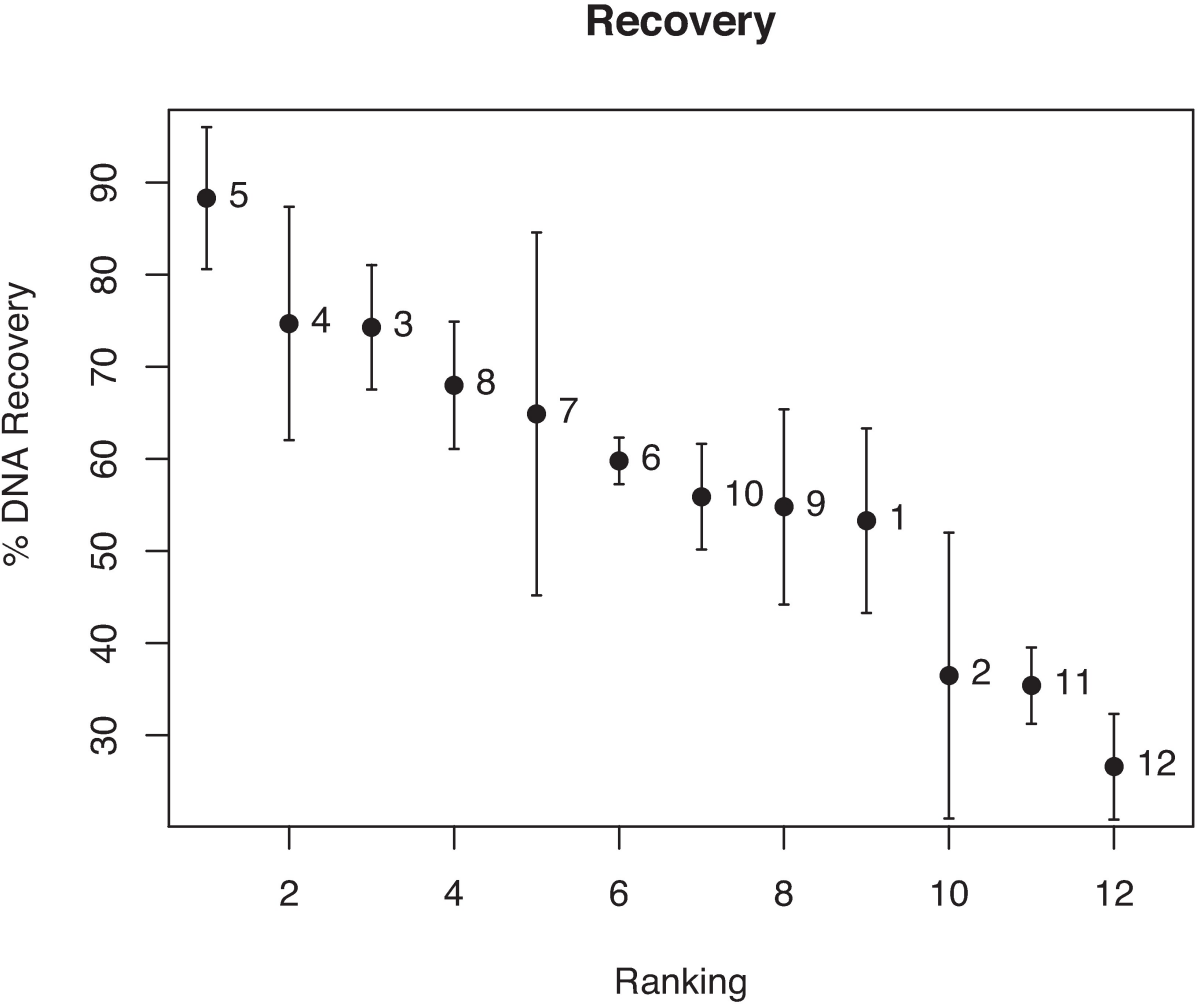
DNA recovery of the twelve bisulfite kits. DNA recovery is shown as percentages ± SD and is calculated by the ratio of the measured output concentration of each bisulfite kit (Qubit ssDNA) to the maximal theoretical output concentration based on the input in every kit (Qubit dsDNA). The labels in the figure refer to the kit numbers in Table 1.

The absolute concentration of the DNA samples was measured on a Qubit 2.0 fluorometer before bisulfite treatment (dsDNA assay) and after bisulfite treatment (ssDNA assay). The starting DNA concentration was on average 136 ng/μl. The DNA recovery ranged from 26.6% (Methyleasy (kit 12)) to 88.3% (EZ Gold (kit 5)) of the maximum theoretical concentration based on the amount of input DNA (Fig 2, S1 and S2 Tables). Of note: different quantification kits (Qubit dsDNA and ssDNA assays) were used before and after bisulfite treatment. The quantification of these two assays was compared by measuring the DNA concentration before and after heat denaturation of a DNA sample. This indicated a similar quantification for both assays (data not shown). Therefore, only a relative comparison of the total DNA loss during the bisulfite conversion of the different kits can be made by this analysis.

### Evaluation of fragmentation

#### Gel electrophoresis

DNA fragmentation by the different bisulfite kits was visualized by gel electrophoresis and Bioanalyzer analysis of the bisulfite treated DNA samples. These methods do not provide quantifiable data about the fragmentation, but provide a rough estimate to compare the best and the worst kits. The untreated gDNA was used as a control showing a clear band of minimally fragmented DNA after the DNA isolation procedure. A smear of larger fragments was detected for the Epitect (kit 10) and CpGenome (kit 11) kits, indicating that these are the least fragmenting kits (Table 1, S1 Fig).

**Table 1:** Overview over the kits and their main characteristics and performance results.

							Recovery	Fragmentation			Conversion
Kit	Name	Short name	Conversion temperature (°C) ^a^	Conversion time (min) ^a^	Input (ng DNA)	Elution volume (μl)	Qubit (ranking)	Gel electrophoresis (integrity: ++ = high;— = low)	qPCR (ranking)	dPCR (ranking)	Conversion efficiency (% ± SD)
**1**	Bisulflash™ DNA Modification Kit (Epigentek, P-1026)	Bisulflash	95	20	350	15	9	-	11	10	Not Assayed
**2**	Bisulflash™ DNA Bisulfite Conversion Easy Kit (Epigentek, P-1054)	Bisulflash Easy	80	45	135.8^b^	15	10	-	12	12	Not Assayed
**3**	Premium Bisulfite Kit (Diagenode, C02030030)	Premium	98 + 54	8 + 60	350	10	3	-	9	9	Not Assayed
**4**	Imprint® DNA Modification Kit (Sigma-Aldrich, MOD50)	Imprint	99 + 65	6 + 90	350	16	2	+/-	8	7	93.2 ± 9.3
**5**	EZ DNA Methylation-Gold™ Kit (Zymo Research, D5005)	EZ Gold	98 + 64	10 + 150	350	10	1	+/-	6	5	99.7 ± 0.1
**6**	EZ DNA Methylation-Lightning™ Kit (Zymo Research, D5030)	EZ Lightning	98 + 54	8 + 60	350	10	6	+	7	8	99.5 ± 0.1
**7**	Fast Bisulfite Conversion Kit (Abcam®, ab1127127)	Fast	95	20	135.8^b^	15	5	-	10	11	Not Assayed
**8**	innuCONVERT Bisulfite Basic kit (Analytic Jena, 845-IC-1000008)	InnuCONVERT	85	45	1500	50	4	+	3	6	99.4 ± 0.4
**9**	Epitect® Fast DNA Bisulfite Kit (Qiagen, 59824)	Epitect Fast	95 +60	10 + 20	1000	15	8	+	3	4	98.0 ± 2.2
**10**	Epitect® Bisulfite Kit (Qiagen, 59110)	Epitect	95 +60	15 + 285	1000	20	7	++	1	2	98.3 ± 0.7
**11**	CpGenome™ Turbo Bisulfite Modification Kit (Merck Millipore, S7847)	CpGenome	37 + 70	10 + 40	500	35	11	++	2	1	No data
**12**	Methyleasy™ Xceed (Human Genetic Signatures, ME001)	Methyleasy	37 + 80	15 + 45	2146^b^	55	12	+	5	3	97.7 ± 2.0

^a^ If two values are given, the pre-incubation step (denaturation) was at another temperature than the incubation step. The first value indicates the pre-incubation temperature or time, the second value indicates the incubation temperature or time.

^b^ Recommended amount of input DNA in the protocol was given in volume. Since the recommended protocol was used, input varied for every donor sample. The input in this table is the average input of the samples from the different donors.

#### qPCR

**Fig 3:**
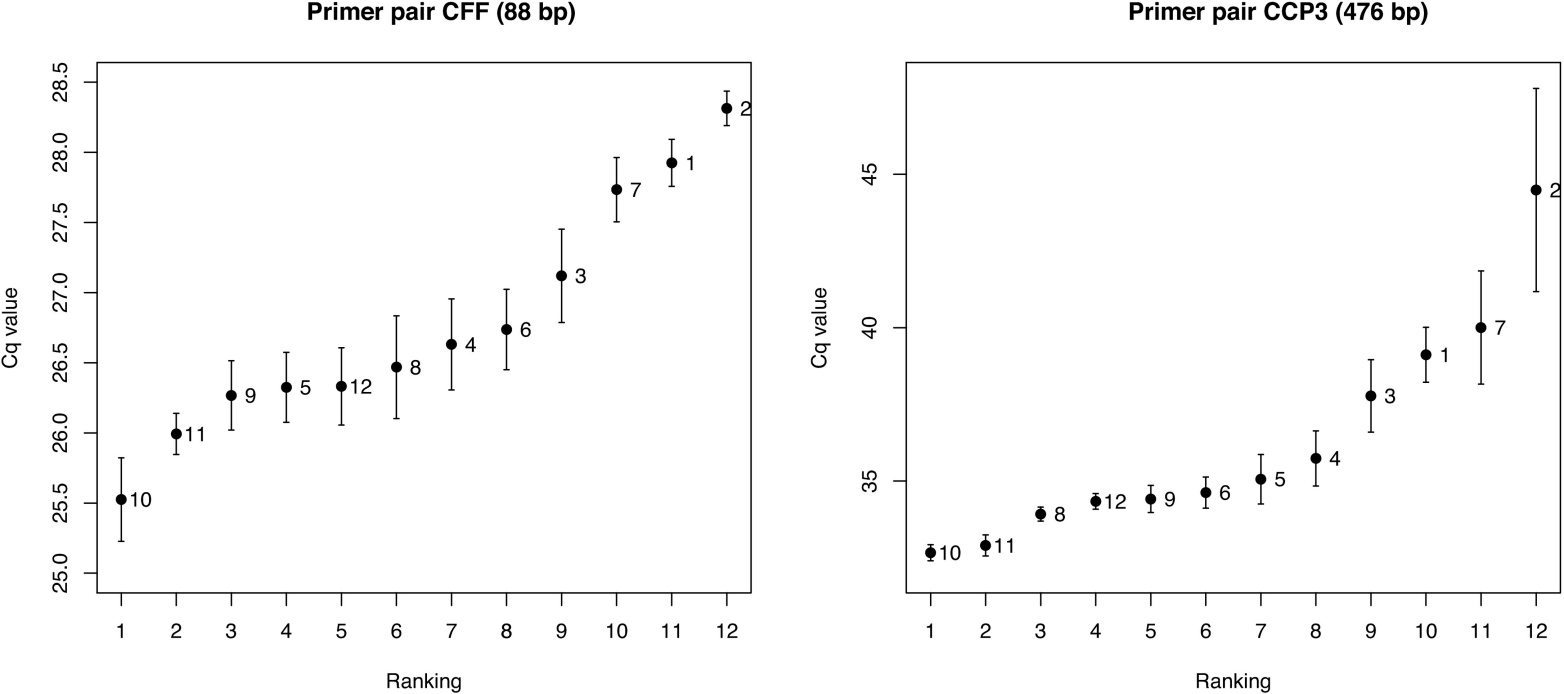
Cq values ± SD of the smallest and the largest amplicons. The data used are the geometric means of the average values from the five donor samples as shown in S3 Table. The data labels refer to the kit number as provided in Table 1.

The comparison of fragmentation of the kits by qPCR included different primer pairs which were developed to amplify amplicons with lengths varying from 88 base pairs (bp) to 476 bp. Overall, the smallest Cq values were found in the Epitect (kit 10) and CpGenome (kit 11) indicating that the concentration of intact DNA strands of the analyzed length is the highest in these kits (Fig 3).

#### Digital PCR

**Fig 4:**
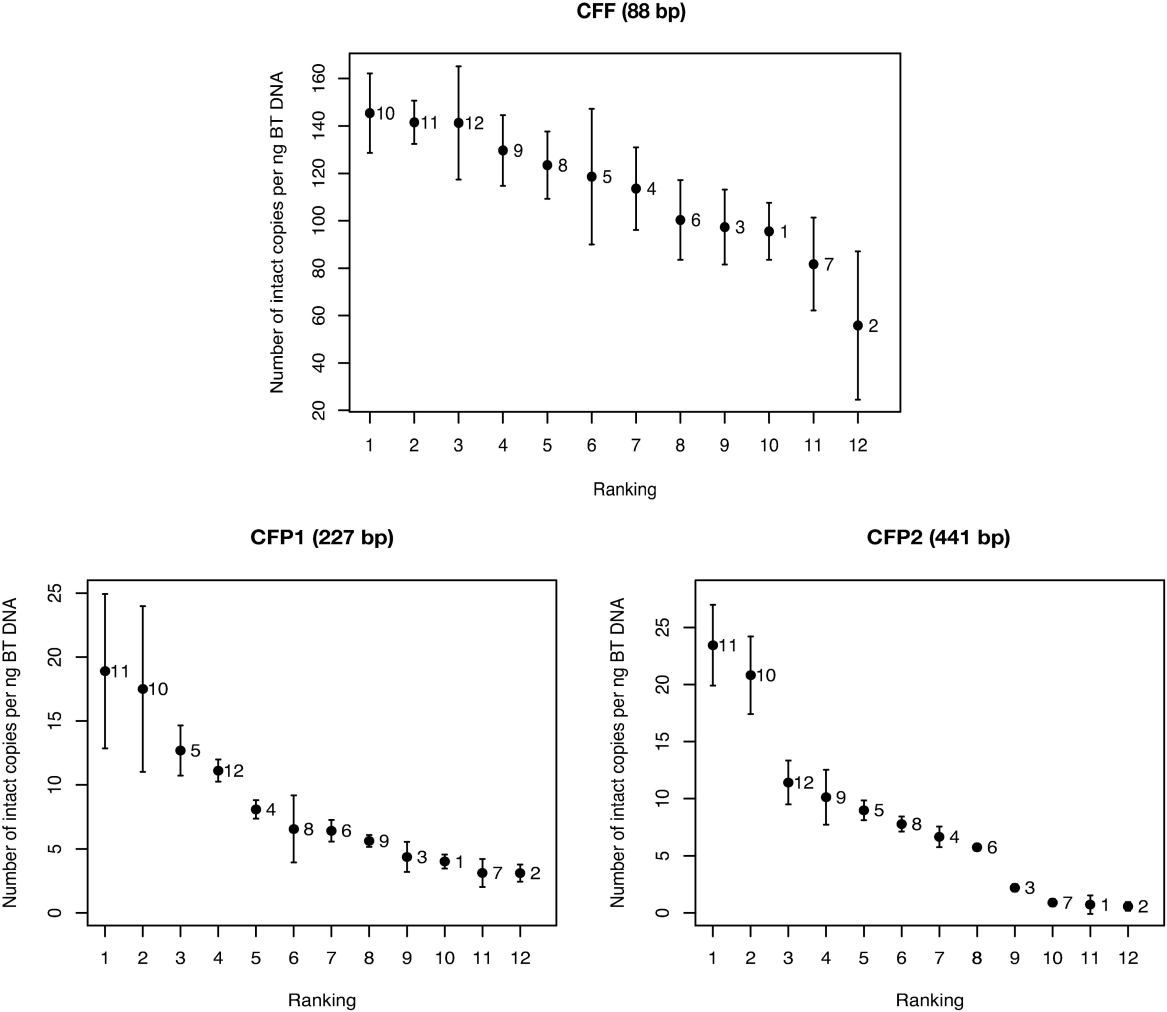
Visual representation of the rankings of the dPCR experiments. Results are given as the geometric means of the average number of intact copies per ng bisulfite treated (BT) DNA measured by dPCR ± SD of all the five donor samples as shown in S4 Table. The data labels refer to the kit number as provided in Table 1.

To test whether digital PCR (dPCR) can be used to assess both DNA recovery as well as DNA fragmentation we used several primers pairs resulting in amplicons of different lengths (88 to 414 bp) to investigate the DNA recovery and the difference in fragmentation between the twelve kits with dPCR. Since dPCR provides direct absolute quantification, the exact amount of DNA strands lost during conversion is measured. Overall, the highest DNA concentrations were found in the Epitect (kit 10) and CpGenome (kit 11): respectively 143.7 and 141.1 intact DNA strands of 88 nt; 16.5 and 17.8 intact DNA strands of 227 nt and 20.8 and 23.3 intact DNA strands of 414 nt per ng input DNA (Fig 4, Table 1 and S4 Table).

Absolute quantification by dPCR before and after treatment showed a significant decrease in number of intact copies in all kits when the length increased from 88 to 227 nt (on average 72.4% DNA lost vs 97.3% DNA lost, Wilcoxon rank sum test, p-value = 7.40e-07), but there was no significant effect of the increase in length from 227 to 414 nt (on average 97.3% DNA lost vs. 96.1% DNA lost, Wilcoxon rank sum test, p-value = 0.478) (S5 Table). Between the different bisulfite kits, the Epitect (kit 10) retained 10.2% of the input DNA with length of 414 nt, while the Bisulflash easy (kit 2) retained only 0.2%. The same trend of DNA recovery (on average over all the kits) was found with the Qubit analysis (see S2 Fig).

### Evaluation of conversion efficiency and inappropriate conversion

Since correct estimation of the methylation in the samples is the main goal after bisulfite treatment, both appropriate conversion (i.e. conversion of C to U) and inappropriate conversion (i.e. conversion of mC to thymine (T)) are crucial characteristics of a bisulfite kit. Therefore, all kits were excluded from the comparison if the appropriate conversion was lower than 95%. This conversion efficiency of the different bisulfite kits was analyzed by the amount of unconverted Cs situated outside CpGs: in principle, 100% conversion of these Cs is expected, since non-CpG Cs are seldom (<0.02%) methylated in human PBMCs [43–48]. Bisulfite treated samples from one donor obtained with eight kits with best scores in the evaluation of the fragmentation and recovery were analyzed (see S6 Table). Two kits (Imprint (kit 4) and CpGenome (kit 11)) showed lower conversion efficiencies for the non-CpG Cs in amplicon CFP2 (62 Cs, 2 CpG) compared to amplicon CCP3 (159 Cs, 32 CpG), indicating that the bisulfite conversion was incomplete. However, the coverage of the sequencing reaction of CpGenome (kit 11) was low (only 14 reads) (S6 Table). Consequently, these kits were excluded from the final ranking. Moreover, a relative comparison of the inappropriate conversion was made based on the methylation percentages obtained with the 34 CpGs analyzed. Gold (kit 5) showed highest methylation (56.2% methylation), indicating that this kit shows the least inappropriate conversion, followed by Epitect (kit 10) (46.4% methylation) and Imprint (kit 4) (42.5% methylation) (S6 Table and S1 Dataset). Of note: as a control, the variability of conversion efficiency between different donors was analyzed. Therefore, the conversion efficiency of samples from a second donor obtained with three kits (innuCONVERT (kit 8), Epitect (kit 10) and Methyleasy (kit 12)) were analyzed and a low variability was observed: standard deviation between 0.01 and 2.42% (data not shown).

### Evaluation of effect of conversion time and temperature

**Fig 5:**
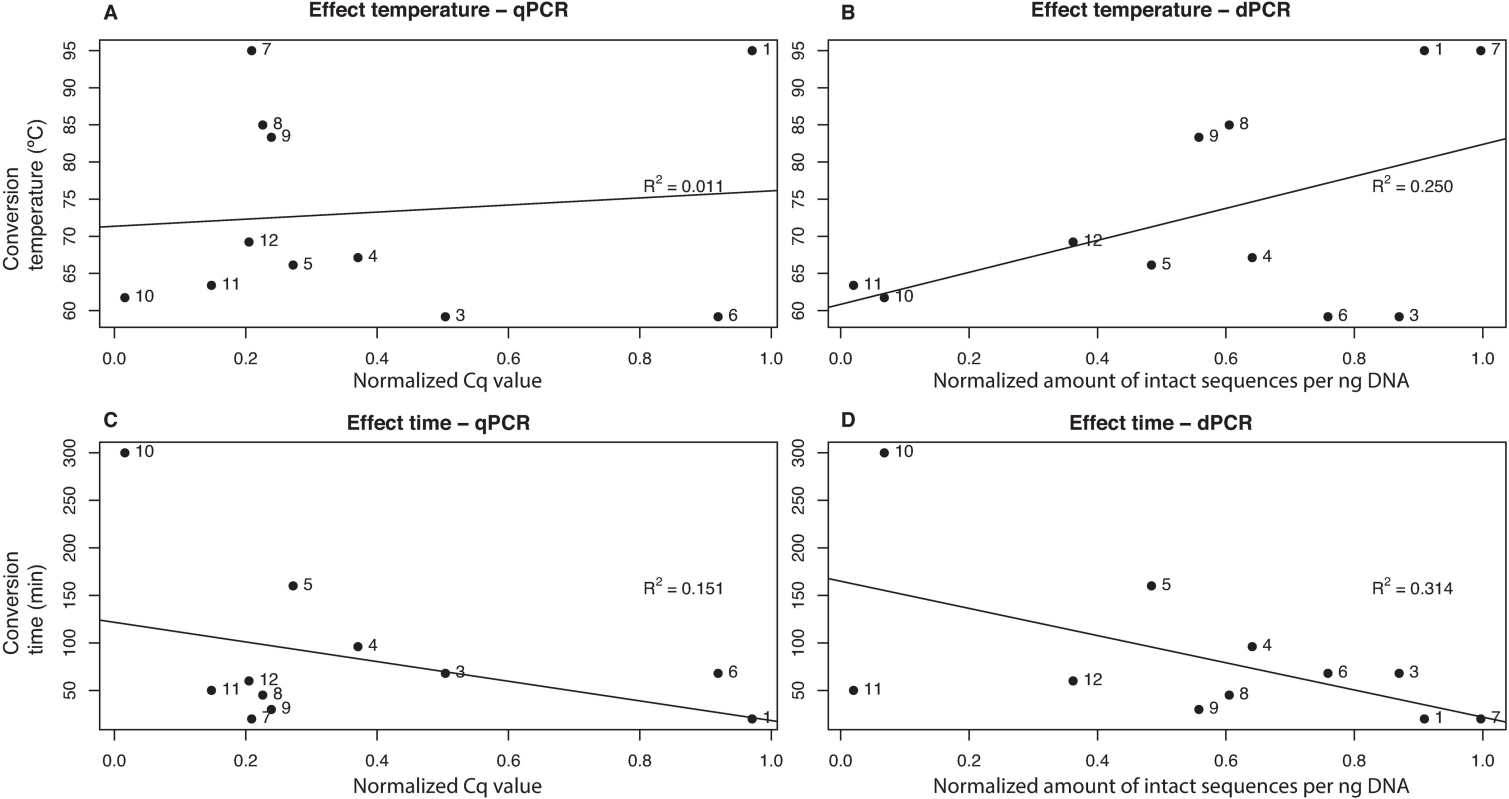
Effect of time and temperature. Effect of time and temperature of the conversion protocol on fragmentation between the different bisulfite conversion kits. Labels refer to the kit numbers in Table 1. The upper panels (A-B) show the effect of different conversion temperatures between the kits measured by qPCR (A) and dPCR (B). The lower panels (C-D) show the effect of different conversion times between the kits measured by qPCR (C) and dPCR (D). The values on the x-axis are normalized to show the relative amount of fragmentation: 0 is least fragmenting and 1 is most fragmenting.

To analyze the effect of increasing temperature and time of the bisulfite conversion on the fragmentation, the degree of fragmentation of the different kits was linked with the time and conversion temperature of each kit (Fig 5). The kits were ranked by fragmentation performance based on the qPCR and dPCR results. No significant correlation between fragmentation and time or temperature was found (Spearman's rank correlation p-values: 0.521 (qPCR–temperature), 0.155 (dPCR–temperature), 0.374 (qPCR–time) and 0.079 (dPCR–time)). To exclusively analyze the effect of conversion temperature and time within a single kit, the fragmentation was compared by changing the conversion temperature and time in Epitect (kit 10). We found no significant difference in fragmentation by these alterations (supplements: S7–S9 Tables and S3 and S4 Figs).

## Discussion

The importance of DNA methylation in different biological processes and the need for an easy-to-use technology have resulted in a wide range of commercially available bisulfite conversion kits. To select the most appropriate bisulfite conversion kit for specific applications, several parameters have to be taken into account. In the current study, we provide a comprehensive workflow for analyzing bisulfite kit performance by comparing twelve bisulfite kits based on DNA recovery and DNA fragmentation. Since dPCR provides a direct absolute quantification with minimal influence of variations in PCR efficiency [49], we can use one method to directly compare samples before and after bisulfite conversion, enabling us to investigate both DNA recovery and fragmentation with more ease and higher accuracy compared to classical methods. The present work shows the strength of dPCR for analysis of the quality of bisulfite treated DNA.

The most essential parameter of a bisulfite kit is the conversion rate, for which appropriate conversion should be maximized, and inappropriate conversion minimized. Our results showed slightly higher estimation of the conversion rates for the cytosine containing (CC) primers (or MethPrimers as called by Fuso et al. [50]) than for the cytosine free (CF) primers (or Methylation Insensitive Primers (MIT) as in the manuscript of Fuso et al. [50]) in seven of the eight samples, which was expected since the reactions with the CF primers amplify both converted as unconverted DNA strands. Since non-CpG methylation is negligible in human PBMCs, it is very unlikely that this bias is similar to the bias described in Fuso et al. [50] which is due to methylated non-CpG Cs. During our comparison, CpGenome (kit 11) and Imprint (kit 4) showed a large difference between the two primer types, indicating a low overall conversion efficiency, and were therefore excluded from the comparison. However, CpGenome (kit 11) had no reliable coverage in the sequencing reaction, for which it also had to be excluded. In other similar studies performed by Holmes et al. [22], Leontiou et al. [39], Izzi et al. [40] and Bryzgunova et al. [41], the conversion efficiency was >95% for all the analyzed kits.

In the current comparison, we showed clear differences between the DNA recovery of the bisulfite kits. This recovery is measured as the overall loss of DNA during the bisulfite treatment, including DNA loss and fragmentation. Remarkably, the best recovery was found with EZ Gold (kit 5), while EZ Lightning (kit 6) was ranked on the sixth place. Since both kits appear to use the same DNA desulfonation and DNA clean up, the conversion buffers and conditions may have impacted the DNA recovery. Our ranking of the recovery of the kits is in accordance with previous comparative studies [22,39–41]. However, in these studies, DNA recovery and fragmentation was never evaluated in depth: Leontiou et al. [39] evaluated both DNA fragmentation and recovery by only measuring DNA concentration via spectroscopic measurements, assuming that fragmentation leads to degraded DNA to such extent that it cannot be analyzed by spectroscopic means. Consequently, our ranking for fragmentation is completely different. Moreover, our findings show that their assumption may not hold for all kits as we observed that kits with low fragmentation can have low recoveries (e.g. CpGenome (kit 11) and Methyleasy (kit 12)).

To get a more detailed insight of how DNA is lost during bisulfite treatment, we used dPCR to distinguish between DNA loss during clean-up and DNA fragmentation during conversion. This method combines the insights obtained by spectroscopic measurements, gel electrophoresis and qPCR to analyze the DNA recovery and the fragmentation. The exact amount of DNA strands lost during the complete bisulfite treatment can be measured by comparing the concentration before and after treatment. By quantifying the exact amount of DNA fragments of different lengths, a fragmentation assessment can be made. The conclusions made by the dPCR about the fragmentation and the DNA loss are in accordance with the results of the Qubit, qPCR and gel electrophoresis, showing the strength of dPCR to combine recovery and fragmentation analysis in a single platform. In this work, we did not evaluate whether the DNA fragmentation and recovery are differently affected by the conversion step and the clean-up step. Future work could help to decouple the effects of both separate parts of bisulfite treatment on the resulting distribution of fragment sizes to completely understand the fragmentation kinetics and further optimize methylation kit performance.

In the current study, Epitect (kit 10) was selected as the most appropriate kit for methylation studies of long DNA fragments since this kit appears to induce least fragmentation. Its conversion time of approximately 300 minutes is much longer than the average (80 minutes). Of note: we could not exclude a potential bias in favor for Epitect (kit 10) since the DNA extraction and bisulfite treatment kits are provided by Qiagen. Earlier studies by Grunau et al., Raizis et al. and Holmes et al. indicated that increased conversion temperature or prolonged conversion time cause more fragmentation [22,32,33]. In the current study, we found no evidence for a significant correlation between time and fragmentation or between temperature and fragmentation for different kits. Interestingly, Grunau et al. found that fragmentation mainly occurs during the first time period of the conversion (after 5 minutes, they observed >90% DNA loss). This could explain the lack of effect on fragmentation by changing the conversion protocol, and it could implicate that the first denaturation step is the most important factor for the fragmentation during conversion. This step was not altered in the alternative protocols of Epitect (kit 10), and could explain the lack of effect on the fragmentation of these alterations. Although, this long incubation could potentially influence the conversion efficiency and it does hamper high throughput methylation analysis with this kit [51]. For high throughput studies, several kits provide a protocol with an incubation time less than 60 minutes. In analysis of shorter fragments, other factors might be decisive: if the amount of DNA is limited, a high DNA recovery is needed, and thus EZ Gold (kit 5) appears to be a better option. Next to this, a kit with a premade bisulfite mix (e.g. EZ Lightning (kit 6)) would fit better to minimize pipetting variation and is ideal for high-throughput analyzes.

In conclusion, our study provides a comprehensive workflow to monitor bisulfite conversion kits based on different parameters such as fragmentation, recovery or conversion efficiency. By using dPCR to analyze the DNA recovery, we are able to analyze the DNA loss and DNA fragmentation with one method. This comprehensive method enables researchers to select the most appropriate kit, depending on the application in which DNA methylation should be analyzed. This test could be important for every study, since the manufacturers can improve the reagents and protocols of the kits over time. In this study, we limited the analysis to standard conditions for the most kits and we only used genomic DNA obtained with one method. Future studies could be performed with equalized DNA input, elution volume, conversion conditions and DNA clean up in order to further understand the dynamics of the bisulfite treatment, or use different types of input DNA in order to assess the influence of the buffers of the extraction method on these parameters. There, our workflow would perfectly suit to be used to monitor the effect of changes during optimizations of a bisulfite kit.

## Material and methods

### Human blood samples

Blood samples from healthy individuals were purchased from the Belgian Red Cross. All donors signed a medical questionnaire containing an informed consent. This consent states that the donated blood can be used for scientific and epidemiologic research if the blood was refused for transfusion. The use of this blood was approved by the Ethical committee of Ghent University Hospital with reference B670201317826.

### DNA samples

Peripheral blood mononuclear cells (PBMCs) from five healthy human donors were isolated using a lymphoprep centrifugation. DNA from aliquots of 10^7^ PBMCs was isolated using the DNeasy^®^ Blood & Tissue Kit (Qiagen, 69504). The DNA samples were all obtained on the same moment, and they were immediately aliquoted into 20 aliquots to ensure the same amount of freeze-thaw cycles for every sample. The DNA concentrations of the samples were determined with the Qubit dsDNA BR (broad range) Assay Kit (Q32850, ThermoFisher Scientific) on a Qubit 2.0 fluorometer, and the exact concentration was verified using the HS (high sensitivity) Assay Kits (Q32851, ThermoFisher Scientific).

### Bisulfite treatment

#### First analysis—selection of the best kits

DNA from the five donor samples was bisulfite treated with twelve different commercially available kits (Table 1). Every sample was converted in duplicate, and after conversion, the duplicates were pooled to provide sufficient material for subsequent testing. For all the kits, the standard protocols provided by the manufacturer with the suggested input/elution volumes were used, and if not exactly provided, the average of the indicated minimal and maximal input/elution were used.

#### Second analysis—effect of time and temperature on fragmentation

Based on the first analysis, the Epitect Bisulfite kit (Qiagen, 59110) was selected as the least fragmenting kit. Subsequently, this kit was used to perform the protocol identically as described above on the five PBMC samples, except for the conversion time or temperature which were modified (S7 and S8 Tables). 1 μg of PBMC DNA from donor 3 (160 ng/μl gDNA) was used, and every conversion was performed in duplicate. The converted DNA was eluted twice in 20 μl elution buffer, and the duplicates were pooled.

### DNA recovery

After conversion, 9 μl of every sample was diluted 1:2 in nuclease free H_2_O. From this 18 μl, 4 μl was used to determine the recovery after each conversion reaction. The Qubit ssDNA Assay Kit (Q10212, ThermoFisher Scientific) was used on a Qubit 2.0 fluorometer to perform duplicate spectrometric measurements on 2 μl of the diluted single-stranded, bisulfite-converted DNA sample.

### Fragmentation

#### Gel electrophoresis

Visualization of the fragmentation was performed by gel electrophoresis using 40 ng converted DNA on an E-Gel^®^ EX Agarose Gel, 2% (Invitrogen, G401002). Since the bisulfite treated DNA was single stranded, and the SYBR Safe dye in the gel only binds dsDNA, a five-minute incubation on an ice bath enabled partial hybridization and visualization of the DNA. The electrophoresis experiments were repeated on an Agilent 2100 Bioanalyzer instrument with an Agilent RNA 6000 Pico Kit.

#### qPCR

To evaluate the amount of intact DNA copies of increasing length after bisulfite treatment, the converted DNA was amplified using qPCR with 6 different primer pairs, resulting in amplicons of increasing length (88–476 bp). Two types of primers were used: cytosine free (CF)-primers and cytosine containing (CC)-primers (S10 Table), which are similar to the Methylation Independent Primers and MethPrimers as previously described by Fuso et al. [50]. The CF-primers target genomic regions without C residues, with the same PCR efficiency before and after treatment. The CC-primers target genomic regions that contain non-CpG C residues. In the bisulfite converted samples, CF-primers amplify both converted and unconverted DNA strands, but the CC-primers can only amplify converted DNA strands.

qPCR was performed using the LightCycler^®^ 480 SYBR Green I Master PCR mix (Roche, 04707516001): 2.5 ng of bisulfite converted DNA was added to the qPCR mix containing SYBR Green Mix (2x) and 500 nM forward and reverse primers, in a final volume of 20 μl. PCR amplification reactions consisted of initial denaturation at 95°C for 10 min, followed by 50 cycles (60 cycles for primer pair CCP1) of denaturation at 95°C for 30 sec, annealing at specific temperature (see S10 Table) for 30 sec and elongation at 72°C for 45 sec, and ended by a melting curve analysis from 72°C to 95°C. Every qPCR was performed in triplicate on all five bisulfite treated samples for each kit. The geometric mean of the Cq values among all replicates, which corresponds with the amount of intact fragments, was used to rank all the kits. If the melting curve analysis was not conclusive, amplicons were analyzed based on length, using a HT DNA 5K chip (CLS760675, Perkin Elmer) on a Labchip® GX (Perkin Elmer) (e.g. S5 and S6 Figs) [52].

#### Digital PCR

To eliminate the potential inhibitory effect of elution buffers or impurities on the qPCR reaction, fragmentation was also analyzed using dPCR. Because of the ‘digital’ end-point measurement in dPCR, effects of PCR efficiencies are eliminated in the quantification. Moreover, with dPCR, it is possible to perform a direct absolute quantification of intact DNA copies in the PCR mix [49,53]. Similar as in the qPCR, several CF primers resulting in amplicons of different lengths (88 to 414 bp), were used to investigate the difference in fragmentation between the twelve kits.

2 μl DNA of all 60 bisulfite treated samples (1:2 diluted) was added to the dPCR mix containing QX200 ddPCR EvaGreen Supermix (2x) (186–4033, Bio-Rad) with 100 nM forward and reverse primers, in a final volume of 20 μl. PCR reactions consisted of initial denaturation at 95°C for 5 min, followed by 40 cycles of denaturation at 95°C for 30 sec and annealing/elongation at specific temperature (S10 Table) for 2 min, and ended with a signal stabilization at 4°C for 5 min and 95°C for 5 min. Each sample was analyzed in duplicate. Moreover, 8.65 μl of untreated DNA samples from all donors was restricted with EcoRI enzyme (Promega, R6011) according to the manufacturer’s instructions. 2μl of this restricted DNA was also used for quantification using the same dPCR mix as the treated DNA samples. Based on the DNA concentration (absolute amount of intact copies per ng bisulfite treated input DNA), a ranking of the 12 kits was made for each primer pair.

### Conversion efficiency

The conversion efficiency was calculated as the amount of converted to non-converted non-CpG Cs, which are usually not methylated in human PBMCs [43–48]. Almost all these Cs should thus be converted during bisulfite treatment, providing us with a method to calculate the conversion efficiency.

Based on the results from the DNA recovery and the fragmentation analysis, we selected the eight least fragmenting kits for subsequent sequencing. We used qPCR amplicons of donor 2 generated with primer pairs CCP3 (149 Cs in amplicon, of which 32 CpG) and CFP2 (62 Cs in amplicon, of which 2 CpG) for subsequent sequencing. The second primer pair can be used to assess the overall conversion efficiency, while analysis with CCP3 can potentially result in an overestimation of this efficiency. To assess the variability between the different donors, converted DNA samples of a second donor (donor 3) obtained by three of the kits were also analyzed. The amplicons were purified with the High Pure PCR Product Purification Kit (11732668001, Roche) and sequenced in a MiSeq sequencing system (Illumina). The sequencing reads were aligned using the Bismark package (version 0.10.1), and further analysis for the conversion efficiency was done by the MethylKit package (version 0.9.5) in R.

## Supporting information

S1 Table Concentration of the DNA samples before bisulfite treatment. The concentration of the untreated DNA samples obtained from PBMCs of five donors before bisulfite treatment measured by Qubit dsDNA BR (broad range) Assay Kit.
(DOCX)

S2 Table Amount of input DNA for bisulfite treatment and concentration of the DNA samples after bisulfite treatment. Two aliquots of all five DNA samples were treated two independent times, yielding in ten bisulfite treated samples, and subsequently, the duplicate samples were pooled. The quantification measurements are done in duplicate with the Qubit ssDNA Assay kit, and the data shown are averages ± SD of these ten concentrations.
(DOCX)

S3 Table Cq values and ranking of the qPCR experiments from the six used primer pairs. First the average of the three technical replicates was calculated for all five samples. The data given is the geometric mean of the average values from the five donor samples. Genomic DNA is the measurement of untreated DNA, which is only conducted for the cytosine free primers since they have the same efficiency before and after treatment. The different kits are ranked by the Cq values for every primer pair (Rank in the table). Subsequently, the median of these rankings is calculated to assess a final ranking. This is the ranking given in Table 1.
(DOCX)

S4 Table Results and ranking of the dPCR experiments from the three primer pairs that were used. First, the average of the two technical replicates was calculated and normalized to the DNA input for all five samples. The data given is the geometric mean of the average values from all five donor samples. The different kits are ranked by the amount of copies per ng bisulfite treated DNA for every primer pair (Rank in the table). Subsequently, the median of these rankings is calculated to assess a final ranking. This is the final ranking given in Table 1.
(DOCX)

S5 Table The percentages of overall DNA loss in the samples. The DNA loss is assessed by dPCR before and after bisulfite treatment. This dPCR measures the intact copies of specific lengths present in the samples. To obtain the averages given in the table, the average of the two technical replicates was calculated both before and after bisulfite treatment. These averages were used to calculate the average loss per kit of all the five donor samples. Subsequently, the geometric means and standard deviations from these averages were calculated.
(DOCX)

S6 Table Conversion efficiencies and overall methylation percentage for the different kits. The data is obtained by sequencing of the 8 kits that performed best in the previous fragmentation assessments. One out of five donor samples was used, and two separate PCR products were sequenced: amplicons CFP2 and CCP3. CFP2 (414 bp) counts 62 Cs, of which 2 CpGs; CCP3 (476 bp) counts 159 Cs, of which 32 CpGs.
(DOCX)

S7 Table Conversion protocol for different temperatures for Epitect (kit 10). The different protocols followed a fixed time schedule as provided by the manual from the manufacturer. Temperature protocol 3 is the same as the protocol provided by the manufacturer.
(DOCX)

S8 Table Conversion protocol for different time schedules for Epitect (kit 10). The different protocols always followed the temperature scheme as provided by the manual from the manufacturer. Time protocol 3 is the same as the protocol provided by the manufacturer.
(DOCX)

S9 Table Concentration after elution with Epitect (kit 10) with the different time and temperature protocols as depicted in S7 and S8 Tables. In the end of the protocol, 2 elutions of the same sample were performed (as recommended by the manufacturer to maximize DNA yield). The data given is a single measurement of the donor used in these time and temperature experiments.
(DOCX)

S10 Table Primer sequences and annealing temperatures for qPCR and dPCR. (DOCX)

S1 Fig **Bioanalyzer (upper plots) and gel electrophoresis (lower plots) analysis of the different kits.** The plots shown in this supplement show the electrophoresis data of the five donor samples.
(PDF)

S2 Fig Comparison of different recovery analyzes (red: Qubit vs black: dPCR). This recovery is based on the amount of overall DNA loss in the samples as shown in S5 Table.
(TIF)

S3 Fig qPCR analysis of the alternative protocols from the Epitect Bisulfite kit as shown in S7 and S8 Tables. Upper panel: protocol changed in conversion temperature (S7 Table). Lower panel: protocol changed in conversion time (S8 Table). Results are given as Cq values ± SD.
(TIF)

S4 Fig dPCR analysis of the alternative protocols from the Epitect Bisulfite kit as shown in S7 and S8 Tables. Upper panel: protocol changed in conversion temperature (S7 Table). Lower panel: protocol changed in conversion time (S8 Table). Results are given as number of intact copies per ng bisulfite treated DNA measured by dPCR ± SD.
(TIF)

S5 Fig Example of the melting curves (upper panel) and the Caliper LabChip GX results (lower panel) after qPCR showing aspecific melting curves, but specific bands for the same reaction: CCP1_after.
(TIF)

S6 Fig Example of the melting curves (upper panel) and the Caliper LabChip GX results (lower panel) after qPCR showing aspecific melting curves, but specific bands for the same reaction: CCP2_after.
(TIF)

S1 Dataset Sequencing data used for the conversion efficiency. Fastq files of the sequencing data that were used for the conversion efficiency calculation.
(ZIP)
