# Supplementary material for: Evaluation of bisulfite kits for DNA methylation profiling in terms of DNA fragmentation and DNA recovery using digital PCR
Source: PLoS One. 2018 Jun 14;13(6):e0199091. doi: 10.1371/journal.pone.0199091 (PMC6002050; doi:10.1371/journal.pone.0199091)
Supplement: S1 Table — The concentration of the untreated DNA samples obtained from PBMCs of five donors before bisulfite treatment measured by Qubit dsDNA BR (broad range) Assay Kit. (DOCX) [file pone.0199091.s001.docx]

**S1 Table. Concentration of the DNA samples before bisulfite treatment.**The concentration of the untreated DNA samples obtained from PBMCs of five donors before bisulfite treatment measured by Qubit dsDNA BR (broad range) Assay Kit.

| Donor | Concentration (ng/µl) |
| --- | --- |
| 1 | 100 |
| 2 | 116 |
| 3 | 160 |
| 4 | 148 |
| 5 | 158 |
