# Supplementary material for: Evaluation of bisulfite kits for DNA methylation profiling in terms of DNA fragmentation and DNA recovery using digital PCR
Source: PLoS One. 2018 Jun 14;13(6):e0199091. doi: 10.1371/journal.pone.0199091 (PMC6002050; doi:10.1371/journal.pone.0199091)
Supplement: S2 Table — Two aliquots of all five DNA samples were treated two independent times, yielding in ten bisulfite treated samples, and subsequently, the duplicate samples were pooled. The quantification measurements are done in duplicate with the Qubit ssDNA Assay kit, and the data shown are averages ± SD of these ten concentrations. (DOCX) [file pone.0199091.s002.docx]

**S2 Table.** **Amount of input DNA for bisulfite treatment and concentration of the DNA samples after bisulfite treatment.**
Two aliquots of all five DNA samples were treated two independent times, yielding in ten bisulfite treated samples, and subsequently, the duplicate samples were pooled. The quantification measurements are done in duplicate with the Qubit ssDNA Assay kit, and the data shown are averages ± SD of these ten concentrations.

| Kit | DNA input (ng) | Elution volume (µl) | Maximal theoretical concentration (ng/µl) | Concentration (ng/µl ± SD) | Recovery (% ± SD) | Ranking |
| --- | --- | --- | --- | --- | --- | --- |
| Bisulflash | 350 | 15 | 23.3 | 12.2 ± 2.4 | 53.3 ± 10.1 | **9** |
| Bisulflash Easy | 135.8^a^ | 15 | 9.1 | 3.22 ± 1.41 | 36.5 ± 15.6 | **10** |
| Premium | 350 | 10 | 35.0 | 26.0 ± 2.4 | 74.3 ± 6.8 | **3** |
| Imprint | 350 | 16 | 21.9 | 16.3 ± 2.8 | 74.7 ± 12.7 | **2** |
| EZ Gold | 350 | 10 | 35.0 | 30.9 ± 2.7 | 88.3 ± 7.7 | **1** |
| EZ Lightning | 350 | 10 | 35.0 | 20.9 ± 0.9 | 59.8 ± 2.6 | **6** |
| Fast | 135.8^a^ | 15 | 9.1 | 5.88 ± 1.78 | 64.9 ± 19.7 | **5** |
| InnuCONVERT | 1500 | 50 | 30.0 | 20.4 ± 2.1 | 68.0 ± 7.0 | **4** |
| Epitect Fast | 1000 | 15 | 66.7 | 36.6 ± 7.1 | 54.8 ± 10.6 | **8** |
| Epitect | 1000 | 20 | 50.0 | 28.0 ± 2.9 | 55.9 ± 5.8 | **7** |
| CpGenome | 500 | 35 | 14.3 | 5.06 ± 0.59 | 35.4 ± 4.2 | **11** |
| Methyleasy | 2146^a^ | 55 | 39.0 | 10.4 ± 2.3 | 26.6 ± 5.8 | **12** |

^a^ Recommended DNA input was given in volume DNA sample. Values are the mean of the actual input
