# Supplementary material for: Evaluation of bisulfite kits for DNA methylation profiling in terms of DNA fragmentation and DNA recovery using digital PCR
Source: PLoS One. 2018 Jun 14;13(6):e0199091. doi: 10.1371/journal.pone.0199091 (PMC6002050; doi:10.1371/journal.pone.0199091)
Supplement: S3 Table — First the average of the three technical replicates was calculated for all five samples. The data given is the geometric mean of the average values from the five donor samples. Genomic DNA is the measurement of untreated DNA, which is only conducted for the cytosine free primers since they have the same efficiency before and after treatment. The different kits are ranked by the Cq values for every primer pair (Rank in the table). Subsequently, the median of these rankings is calculated to assess a final ranking. This is the ranking given in Table 1. (DOCX) [file pone.0199091.s003.docx]

**S3 Table. Cq values and ranking of the qPCR experiments from the six used primer pairs.**
First the average of the three technical replicates was calculated for all five samples. The data given is the geometric mean of the average values from the five donor samples. Genomic DNA is the measurement of untreated DNA, which is only conducted for the cytosine free primers since they have the same efficiency before and after treatment. The different kits are ranked by the Cq values for every primer pair (Rank in the table). Subsequently, the median of these rankings is calculated to assess a final ranking. This is the ranking given in Table 1.

| **Kit** | **CFF**  **(Cq ± SD)** | **Rank CFF** | **CFP1**  **(Cq ± SD)** | **Rank CFP1** | **CFP2**  **(Cq ± SD)** | **Rank CFP2** | **CCP1**  **(Cq ± SD)** | **Rank CCP1** | **CCP2**  **(Cq ± SD)** | **Rank CCP2** | **CCP3**  **(Cq ± SD)** | **Rank CCP3** | **Median Rank** | **Final Rank** |
| --- | --- | --- | --- | --- | --- | --- | --- | --- | --- | --- | --- | --- | --- | --- |
| **Bisulflash** | 27.92 ± 0.20 | 11 | 29.69 ± 0.12 | 11 | 37.60 ± 0.83 | 10 | 50.24 ± 1.66 | 11 | 38.68 ± 1.37 | 11 | 39.06 ± 2.30 | 10 | 11 | **11** |
| **Bisulflash Easy** | 28.31 ± 0.23 | 12 | 30.07 ± 0.11 | 12 | 48.96 ± 2.24 | 12 | 57.19 ± 3.90 | 12 | 39.25 ± 1.43 | 12 | 44.40 ± 3.04 | 12 | 12 | **12** |
| **Premium** | 27.12 ± 0.13 | 9 | 28.75 ± 0.18 | 9 | 34.39 ± 0.40 | 8 | 43.35 ± 1.57 | 8 | 34.47 ± 0.47 | 9 | 37.77 ± 0.62 | 9 | 9 | **9** |
| **Imprint** | 26.63 ± 0.23 | 7 | 28.12 ± 0.21 | 8 | 34.23 ± 2.56 | 7 | 43.70 ± 2.81 | 9 | 32.72 ± 0.32 | 8 | 35.73 ± 0.70 | 8 | 8 | **8** |
| **EZ Gold** | 26.32 ± 0.25 | 4 | 28.00 ± 0.27 | 6 | 32.16 ± 0.24 | 2 | 40.85 ± 1.11 | 4 | 32.37 ± 0.39 | 7 | 35.05 ± 0.95 | 7 | 5 | **6** |
| **EZ Lightning** | 26.74 ± 0.24 | 8 | 28.08 ± 0.21 | 7 | 33.55 ± 1.21 | 5 | 41.30 ± 1.00 | 7 | 31.56 ± 0.48 | 5 | 34.63 ± 0.16 | 6 | 6,7 | **7** |
| **Fast** | 27.73 ± 0.16 | 10 | 29.43 ± 0.21 | 10 | 38.16 ± 0.61 | 11 | 47.31 ± 1.17 | 10 | 38.18 ± 0.99 | 10 | 39.98 ± 1.61 | 11 | 10 | **10** |
| **InnuCONVERT** | 26.47 ± 0.18 | 6 | 27.89 ± 0.13 | 5 | 32.81 ± 0.48 | 3 | 41.01 ± 0.95 | 6 | 31.24 ± 0.21 | 3 | 33.93 ± 0.25 | 3 | 4 | **3^a^** |
| **Epitect Fast** | 26.27 ± 0.29 | 3 | 27.85 ± 0.22 | 4 | 33.40 ± 1.82 | 4 | 40.87 ± 1.17 | 5 | 31.55 ± 0.41 | 4 | 34.42 ± 0.46 | 5 | 4 | **3^a^** |
| **Epitect** | 25.52 ± 0.39 | 1 | 27.14 ± 0.26 | 2 | 31.28 ± 1.09 | 1 | 39.68 ± 1.61 | 1 | 30.40 ± 0.39 | 1 | 32.67 ± 0.21 | 1 | 1 | **1** |
| **CpGenome** | 25.99 ± 0.13 | 2 | 26.81 ± 0.26 | 1 | 36.16 ± 0.67 | 9 | 39.72 ± 0.73 | 2 | 30.80 ± 0.33 | 2 | 32.90 ± 0.13 | 2 | 2 | **2** |
| **Methyleasy** | 26.33 ± 0.31 | 5 | 27.56 ± 0.20 | 3 | 33.89 ± 1.69 | 6 | 39.94 ± 1.50 | 3 | 31.71 ± 0.38 | 6 | 34.34 ± 0.34 | 4 | 4,5 | **5** |
| **Genomic DNA** | 20,74 ± 0,17 |  | 21,79 ± 0,13 |  | 23,93 ± 0,58 |  | NA |  | NA |  | NA |  |  |  |

^a^ The same overall ranking was given if two kits obtained the same median of rankings.
