# Supplementary material for: Evaluation of bisulfite kits for DNA methylation profiling in terms of DNA fragmentation and DNA recovery using digital PCR
Source: PLoS One. 2018 Jun 14;13(6):e0199091. doi: 10.1371/journal.pone.0199091 (PMC6002050; doi:10.1371/journal.pone.0199091)
Supplement: S4 Table — First, the average of the two technical replicates was calculated and normalized to the DNA input for all five samples. The data given is the geometric mean of the average values from all five donor samples. The different kits are ranked by the amount of copies per ng bisulfite treated DNA for every primer pair (Rank in the table). Subsequently, the median of these rankings is calculated to assess a final ranking. This is the final ranking given in Table 1. (DOCX) [file pone.0199091.s004.docx]

**S4 Table. Results and ranking of the dPCR experiments from the three primer pairs that were used.**
First, the average of the two technical replicates was calculated and normalized to the DNA input for all five samples. The data given is the geometric mean of the average values from all five donor samples. The different kits are ranked by the amount of copies per ng bisulfite treated DNA for every primer pair (Rank in the table). Subsequently, the median of these rankings is calculated to assess a final ranking. This is the final ranking given in Table 1.

| Kit | CFF (copies per ng DNA ± SD) | Rank  CFF | CFP1 (copies per ng DNA ± SD) | Rank CFP1 | CFP2 (copies per ng DNA ± SD) | Rank  CFP2 | Median  Rank | Final  Rank |
| --- | --- | --- | --- | --- | --- | --- | --- | --- |
| Bisulflash | 92.5 ± 12.1 | 10 | 3.4 ± 0.7 | 10 | 0.7 ± 0.9 | 11 | 10 | **10** |
| Bisulflash Easy | 51.7 ± 31.3 | 12 | 3.0 ± 0.7 | 12 | 0.6 ± 0.4 | 12 | 12 | **12** |
| Premium | 96.5 ± 15.9 | 9 | 4.1 ± 1.3 | 9 | 2.2 ± 0.4 | 9 | 9 | **9** |
| Imprint | 112.7 ± 17.5 | 7 | 7.4 ± 0.9 | 5 | 6.5 ± 1.0 | 7 | 7 | **7** |
| EZ Gold | 117.1 ± 28.7 | 6 | 12.2 ± 3.0 | 3 | 9.0 ± 0.9 | 5 | 5 | **5** |
| EZ Lightning | 98.6 ± 16.8 | 8 | 5.5 ± 0.9 | 7 | 5.7 ± 0.4 | 8 | 8 | **8** |
| Fast | 80.3 ± 19.7 | 11 | 3.0 ± 1.4 | 11 | 0.8 ± 0.4 | 10 | 11 | **11** |
| InnuCONVERT | 122.7 ± 14.3 | 5 | 5.8 ± 2.7 | 6 | 7.6 ± 0.7 | 6 | 6 | **6** |
| Epitect Fast | 129.0 ± 15.0 | 4 | 5.1 ± 0.7 | 8 | 10.0 ± 2.5 | 4 | 4 | **4** |
| Epitect | 143.7 ± 16.8 | 1 | 16.5 ± 7.0 | 2 | 20.8 ± 3.4 | 2 | 2 | **2** |
| CpGenome | 141.1 ± 9.2 | 2 | 17.8 ± 6.7 | 1 | 23.3 ± 3.6 | 1 | 1 | **1** |
| Methyleasy | 138.8 ± 24.0 | 3 | 10.6 ± 1.3 | 4 | 11.3 ± 2.0 | 3 | 3 | **3** |
