# Supplementary material for: Evaluation of bisulfite kits for DNA methylation profiling in terms of DNA fragmentation and DNA recovery using digital PCR
Source: PLoS One. 2018 Jun 14;13(6):e0199091. doi: 10.1371/journal.pone.0199091 (PMC6002050; doi:10.1371/journal.pone.0199091)
Supplement: S5 Table — The DNA loss is assessed by dPCR before and after bisulfite treatment. This dPCR measures the intact copies of specific lengths present in the samples. To obtain the averages given in the table, the average of the two technical replicates was calculated both before and after bisulfite treatment. These averages were used to calculate the average loss per kit of all the five donor samples. Subsequently, the geometric means and standard deviations from these averages were calculated. (DOCX) [file pone.0199091.s005.docx]

**S5 Table. The percentages of overall DNA loss in the samples.**
The DNA loss is assessed by dPCR before and after bisulfite treatment. This dPCR measures the intact copies of specific lengths present in the samples. To obtain the averages given in the table, the average of the two technical replicates was calculated both before and after bisulfite treatment. These averages were used to calculate the average loss per kit of all the five donor samples. Subsequently, the geometric means and standard deviations from these averages were calculated.

| Kit | Average loss intact copies CFF (88 bp) (% ± SD) | Average loss intact copies CFP1 (227 bp) (% ± SD) | Average loss intact copies CFP2 (414 bp) (% ± SD) |
| --- | --- | --- | --- |
| Bisulflash | 79.4 ± 5.2 | 98.8 ± 0.9 | 99.7 ± 0.1 |
| Bisulflash Easy | 91.2 ± 4.4 | 99.4 ± 0.2 | 99.8 ± 0.2 |
| Premium | 69.7 ± 4.8 | 98.1 ± 1.0 | 98.6 ± 0.3 |
| Imprint | 65.0 ± 4.1 | 96.6 ± 1.8 | 95.8 ± 1.2 |
| EZ Gold | 56.3 ± 5.8 | 93.6 ± 2.3 | 93.2 ± 1.2 |
| EZ Lightning | 74.8 ± 5.3 | 97.7 ± 1.6 | 97.1 ± 0.6 |
| Fast | 77.5 ± 4.9 | 98.9 ± 0.3 | 99.5 ± 0.2 |
| InnuCONVERT | 64.9 ± 4.7 | 97.5 ± 1.6 | 95.5 ± 1.4 |
| Epitect Fast | 69.8 ± 2.5 | 98.3 ± 0.8 | 95.2 ± 1.3 |
| Epitect | 65.0 ± 5.2 | 94.3 ± 2.1 | 89.8 ± 1.3 |
| CpGenome | 78.4 ± 2.5 | 96.8 ± 2.2 | 92.8 ± 0.6 |
| Methyleasy | 83.9 ± 4.0 | 98.2 ± 0.9 | 97.3 ± 1.1 |
| Average loss per primer pair | **72.4 ± 9.7** | **97.3 ± 1.8** | **96.1 ± 3.2** |
