# Supplementary material for: Evaluation of bisulfite kits for DNA methylation profiling in terms of DNA fragmentation and DNA recovery using digital PCR
Source: PLoS One. 2018 Jun 14;13(6):e0199091. doi: 10.1371/journal.pone.0199091 (PMC6002050; doi:10.1371/journal.pone.0199091)
Supplement: S6 Table — The data is obtained by sequencing of the 8 kits that performed best in the previous fragmentation assessments. One out of five donor samples was used, and two separate PCR products were sequenced: amplicons CFP2 and CCP3. CFP2 (414 bp) counts 62 Cs, of which 2 CpGs; CCP3 (476 bp) counts 159 Cs, of which 32 CpGs. (DOCX) [file pone.0199091.s006.docx]

**S6 Table. Conversion efficiencies and overall methylation percentage for the different kits.**
The data is obtained by sequencing of the 8 kits that performed best in the previous fragmentation assessments. One out of five donor samples was used, and two separate PCR products were sequenced: amplicons CFP2 and CCP3. CFP2 (414 bp) counts 62 Cs, of which 2 CpGs; CCP3 (476 bp) counts 159 Cs, of which 32 CpGs.

|  | Conversion efficiency | | | Overall methylation | | | Coverage | | |
| --- | --- | --- | --- | --- | --- | --- | --- | --- | --- |
| Kit | CFP2 (%) | CCP3_after (%) | Mean (% ± SD) | CFP2 (%) | CCP3_after (%) | CFP2 | | CCP3_after |  |
| Imprint | 86.6 | 99.7 | 93.2 ± 9.3 | 92.6 | 39.4 | 678 | | 117 |  |
| EZ Gold | 99.7 | 99.6 | 99.7 ± 0.1 | 90.7 | 54.0 | 464 | | 111 |  |
| EZ Lightning | 99.5 | 99.5 | 99.5 ± 0.1 | 93.2 | 26.0 | 659 | | 126 |  |
| InnuCONVERT | 99.2 | 99.7 | 99.4 ± 0.4 | 92.1 | 38.8 | 482 | | 123 |  |
| Epitect Fast | 96.5 | 99.5 | 98.0 ± 2.2 | 91.2 | 32.3 | 952 | | 118 |  |
| Epitect | 98.1 | 98.5 | 98.3 ± 0.7 | 95.1 | 43.4 | 314 | | 42 |  |
| CpGenome | 52.1 | 99.8 | 75.9 ± 33.8 | 100 | 14.3 | 14 | | 14 |  |
| Methyleasy | 96.0 | 99.4 | 97.7 ± 2.0 | 91.3 | 31.5 | 288 | | 62 |  |

_after: primer targeting bisulfite converted DNA
