# Supplementary material for: Evaluation of bisulfite kits for DNA methylation profiling in terms of DNA fragmentation and DNA recovery using digital PCR
Source: PLoS One. 2018 Jun 14;13(6):e0199091. doi: 10.1371/journal.pone.0199091 (PMC6002050; doi:10.1371/journal.pone.0199091)
Supplement: S7 Table — The different protocols followed a fixed time schedule as provided by the manual from the manufacturer. Temperature protocol 3 is the same as the protocol provided by the manufacturer. (DOCX) [file pone.0199091.s007.docx]

**S7 Table. Conversion protocol for different temperatures for Epitect (kit 10).**The different protocols followed a fixed time schedule as provided by the manual from the manufacturer. Temperature protocol 3 is the same as the protocol provided by the manufacturer.

| Step | Time (min) | Temperature protocol 1 (°C) | Temperature protocol 2 (°C) | Temperature protocol 3 (°C) | Temperature protocol 4 (°C) |
| --- | --- | --- | --- | --- | --- |
| Denaturation1 | 5 | 95 | 95 | 95 | 95 |
| Conversion1 | 25 | 40 | 50 | 60 | 75 |
| Denaturation2 | 5 | 95 | 95 | 95 | 95 |
| Conversion2 | 85 | 40 | 50 | 60 | 75 |
| Denaturation3 | 5 | 95 | 95 | 95 | 95 |
| Conversion3 | 175 | 40 | 50 | 60 | 75 |
