# Supplementary material for: Evaluation of bisulfite kits for DNA methylation profiling in terms of DNA fragmentation and DNA recovery using digital PCR
Source: PLoS One. 2018 Jun 14;13(6):e0199091. doi: 10.1371/journal.pone.0199091 (PMC6002050; doi:10.1371/journal.pone.0199091)
Supplement: S8 Table — The different protocols always followed the temperature scheme as provided by the manual from the manufacturer. Time protocol 3 is the same as the protocol provided by the manufacturer. (DOCX) [file pone.0199091.s008.docx]

**S8 Table. Conversion protocol for different time schedules for Epitect (kit 10).**The different protocols always followed the temperature scheme as provided by the manual from the manufacturer. Time protocol 3 is the same as the protocol provided by the manufacturer.

| Step | Temp (°C) | Time protocol 1 (min) | Time protocol 2 (min) | Time protocol 3 (min) | Time protocol 4 (min) |
| --- | --- | --- | --- | --- | --- |
| Denaturation1 | 95 | 5 | 5 | 5 | 5 |
| Conversion1 | 60 | 10 | 20 | 25 | 35 |
| Denaturation2 | 5 | 5 | 5 | 5 | 5 |
| Conversion2 | 60 | 50 | 75 | 85 | 100 |
| Denaturation3 | 5 | 5 | 5 | 5 | 5 |
| Conversion3 | 60 | 100 | 135 | 175 | 200 |
