# Supplementary material for: Evaluation of bisulfite kits for DNA methylation profiling in terms of DNA fragmentation and DNA recovery using digital PCR
Source: PLoS One. 2018 Jun 14;13(6):e0199091. doi: 10.1371/journal.pone.0199091 (PMC6002050; doi:10.1371/journal.pone.0199091)
Supplement: S9 Table — In the end of the protocol, 2 elutions of the same sample were performed (as recommended by the manufacturer to maximize DNA yield). The data given is a single measurement of the donor used in these time and temperature experiments. (DOCX) [file pone.0199091.s009.docx]

**S9 Table. Concentration after elution with Epitect (kit 10) with the different time and temperature protocols as depicted in S7 and S8 Tables.**
In the end of the protocol, 2 elutions of the same sample were performed (as recommended by the manufacturer to maximize DNA yield). The data given is a single measurement of the donor used in these time and temperature experiments.

| protocol | elution1 (ng/µl) | elution2 (ng/µl) | % Recovery (elution1) |
| --- | --- | --- | --- |
| Time 1 (160') | 33.4 | 9.48 | 66.8 |
| Time 2 (230') | 34.0 | 8.56 | 68.0 |
| Time 3^a^ (285') | 34.0 | 10.6 | 68.0 |
| Time 4 (335') | 24.2 | 14.3 | 48.4 |
| Temperature 1 (40°C) | 22.2 | 8.66 | 44.4 |
| Temperature 2(50°C) | 29.8 | 7.84 | 59.6 |
| Temperature 3^a^ (60°C) | 34.0 | 10.6 | 68.0 |
| Temperature 4 (75°C) | 35.0 | 6.20 | 70.0 |

^a^Time3 and Temperature 3 are the standard protocol as provided by the manufacturer.
