# Supplementary material for: Evaluation of bisulfite kits for DNA methylation profiling in terms of DNA fragmentation and DNA recovery using digital PCR
Source: PLoS One. 2018 Jun 14;13(6):e0199091. doi: 10.1371/journal.pone.0199091 (PMC6002050; doi:10.1371/journal.pone.0199091)
Supplement: S10 Table — (DOCX) [file pone.0199091.s010.docx]

**S10 Table. Primer sequences and annealing temperatures for qPCR and dPCR.**

| Primer pair  (amplicon length) | Sequence | Binding region (Ensembl assembly GRCh37) | Annealing temperature qPCR (°C) | Annealing temperature ddPCR (°C) |
| --- | --- | --- | --- | --- |
| CFF (88 bp) | F:TAAGAGTAATAATGGATGGATGATG  R:CCTCCCATCTCCCTTCC | Chr13 : 19555120  Chr13 : 19555208 | 58^a^ – 57^b^ | 58^a^ – 59^b^ |
| CFP1 (227 bp) | F:TGGGTTAAAGTGATTGAGTAA  R:TATTCATCCTTCAACTTACCCT | Chr2 : 21454728  Chr2 : 21454955 | 52^b^ – 53^a^ | 53^b^ – 54^a^ |
| CFP2 (414 bp) | F:ATGGGTAAGGATATGAAGTTAAT  R:TATCACTTAATCACCTCCTAAACTA | Chr2 : 21557568  Chr2 : 21557982 | 51^a^ – 60^b^ | 54^b^ – 56^a^ |
| CCP1_before (175 bp) | F:GCTGAGGGGCAGAGGGAAGTGC  R:GTCTTCAGACAGGAAAGTGGCC | Chr11 : 2019526  Chr11 : 2019701 | 63 – 64 | 65 – 66 |
| CCP2_before (361 bp) | F:GCTGAGGGGCAGAGGGAAGTGC  R:CTCACCAAAGGCCAAGGTGGTGACC | Chr11 : 2019526  Chr11 : 2019887 | 63 | 66 |
| CCP3_before (476 bp) | F:TGCACATGGCTGGGGGCCAGCTG  R:CCCTCACCAAAGGCCAAGGTGGTGAC | Chr11 : 2019413  Chr11 : 2019889 | 64 | 66 |
| CCP1_after (175 bp) | F:GTTGAGGGGTAGAGGGAAGTGT  R:ATCTTCAAACAAAAAAATAACC | Chr11 : 2019526  Chr11 : 2019701 | 60 | / |
| CCP2_after (361 bp) | F:GTTGAGGGGTAGAGGGAAGTGT  R:CTCACCAAAAACCAAAATAATAACC | Chr11 : 2019526  Chr11 : 2019887 | 62 | / |
| CCP3_after (476 bp) | F:TGTATATGGTTGGGGGTTAGTTG  R:CCCTCACCAAAAACCAAAATAATAAC | Chr11 : 2019413  Chr11 : 2019889 | 63 | / |

^a^ Optimal annealing temperature for DNA before bisulfite treatment

^b^ Optimal annealing temperature for DNA after bisulfite treatment

F: Forward primer

R: Reverse primer

_before: primer targeting genomic DNA before bisulfite treatment

_after: primer targeting bisulfite converted DNA
