# Supplementary figures and images for: Evaluation of bisulfite kits for DNA methylation profiling in terms of DNA fragmentation and DNA recovery using digital PCR
Source: PLoS One. 2018 Jun 14;13(6):e0199091. doi: 10.1371/journal.pone.0199091 (PMC6002050; doi:10.1371/journal.pone.0199091)

D1

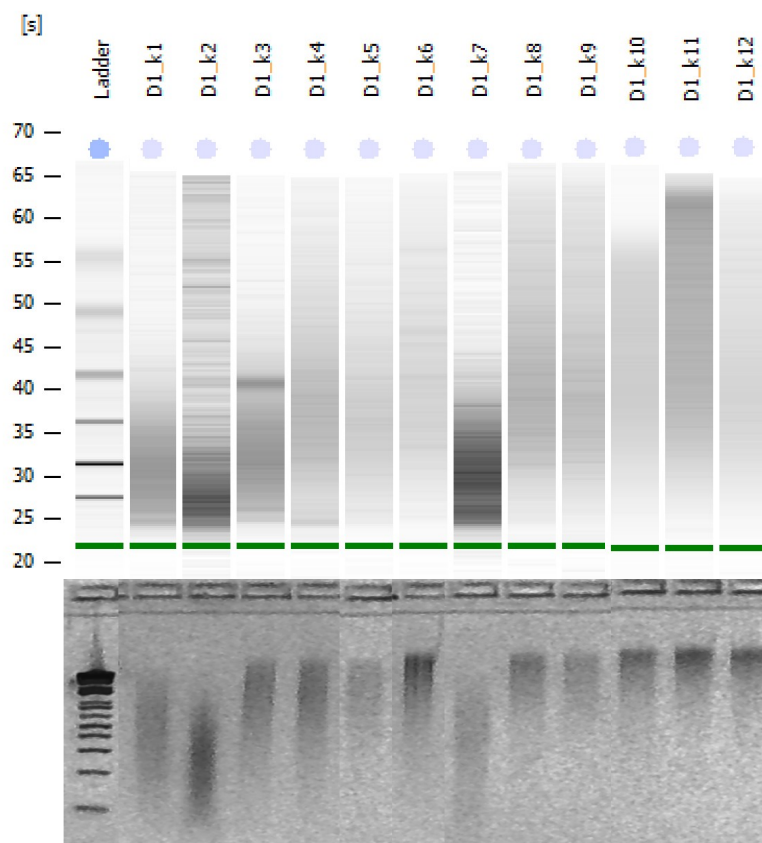

D2

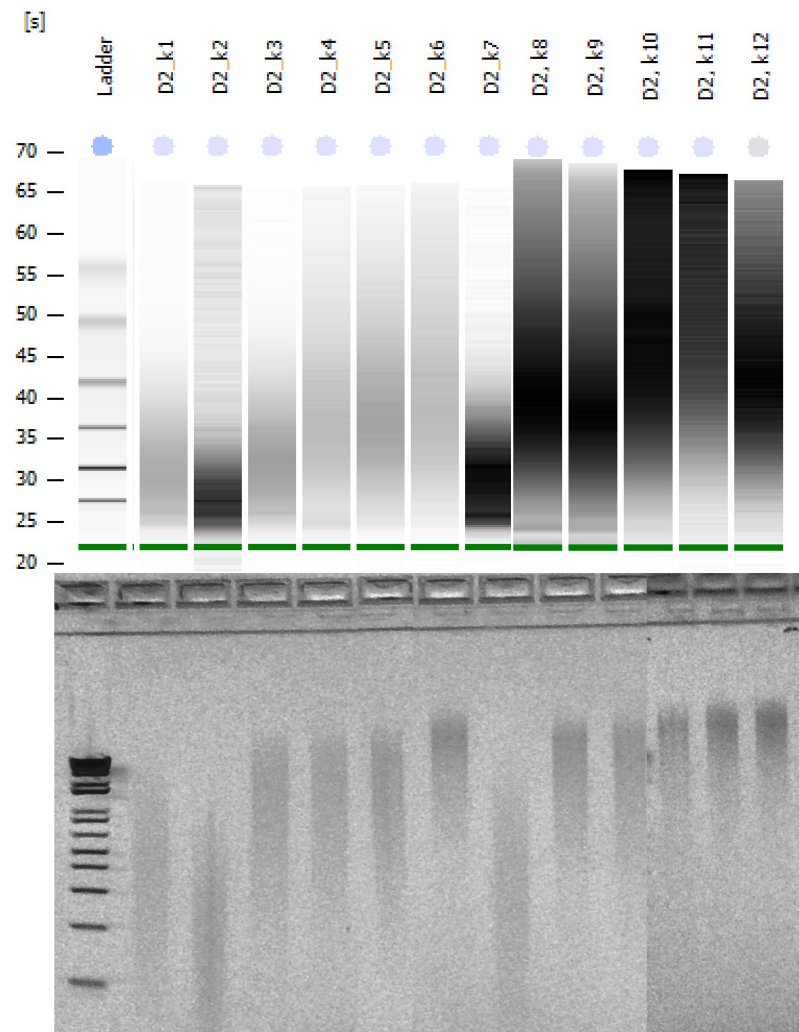

D3

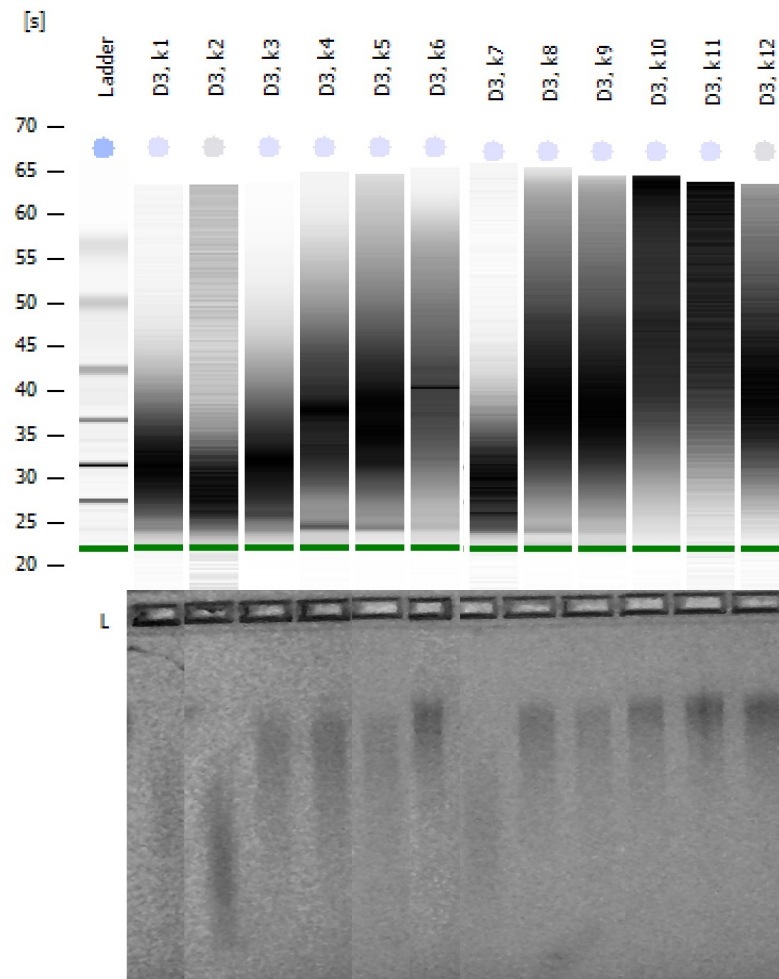

D4

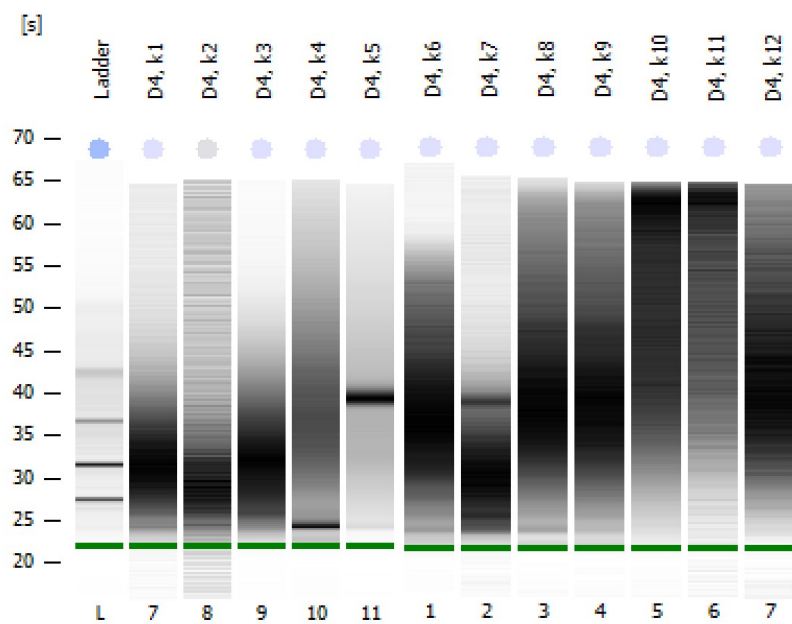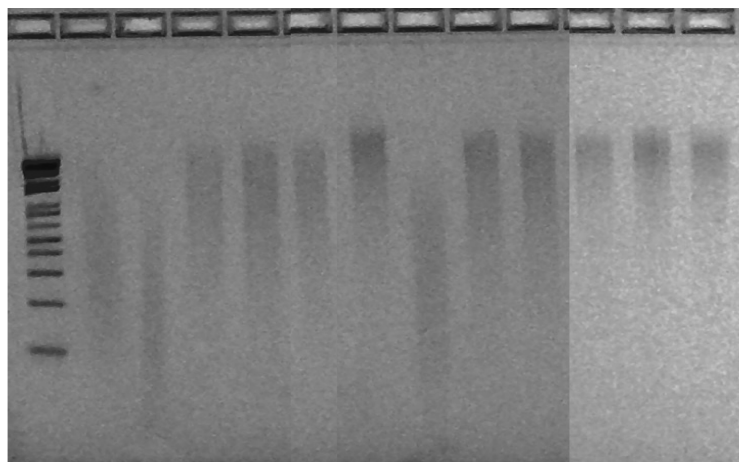

D5

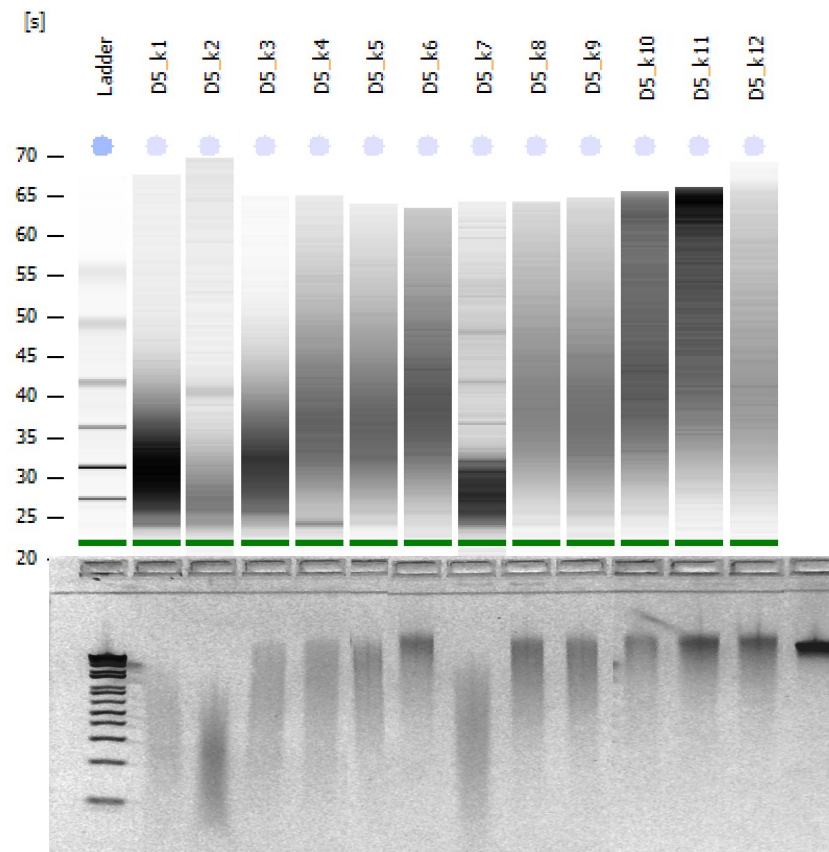

Supplement: S1 Fig — Bioanalyzer (upper plots) and gel electrophoresis (lower plots) analysis of the different kits. The plots shown in this supplement show the electrophoresis data of the five donor samples. (PDF) [file pone.0199091.s011.pdf]

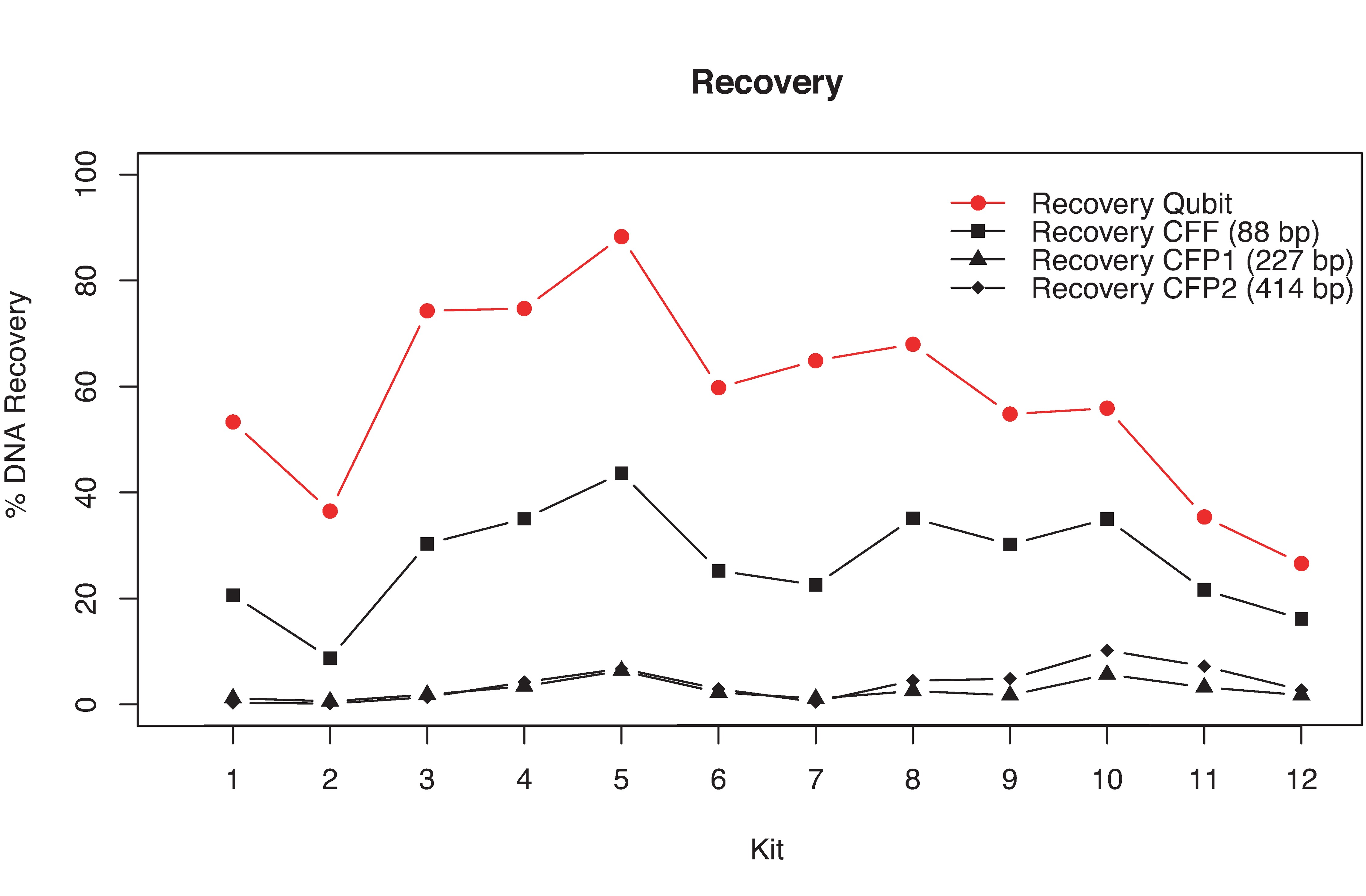

Supplement: S2 Fig — This recovery is based on the amount of overall DNA loss in the samples as shown in S5 Table. (TIF) [file pone.0199091.s012.tif]

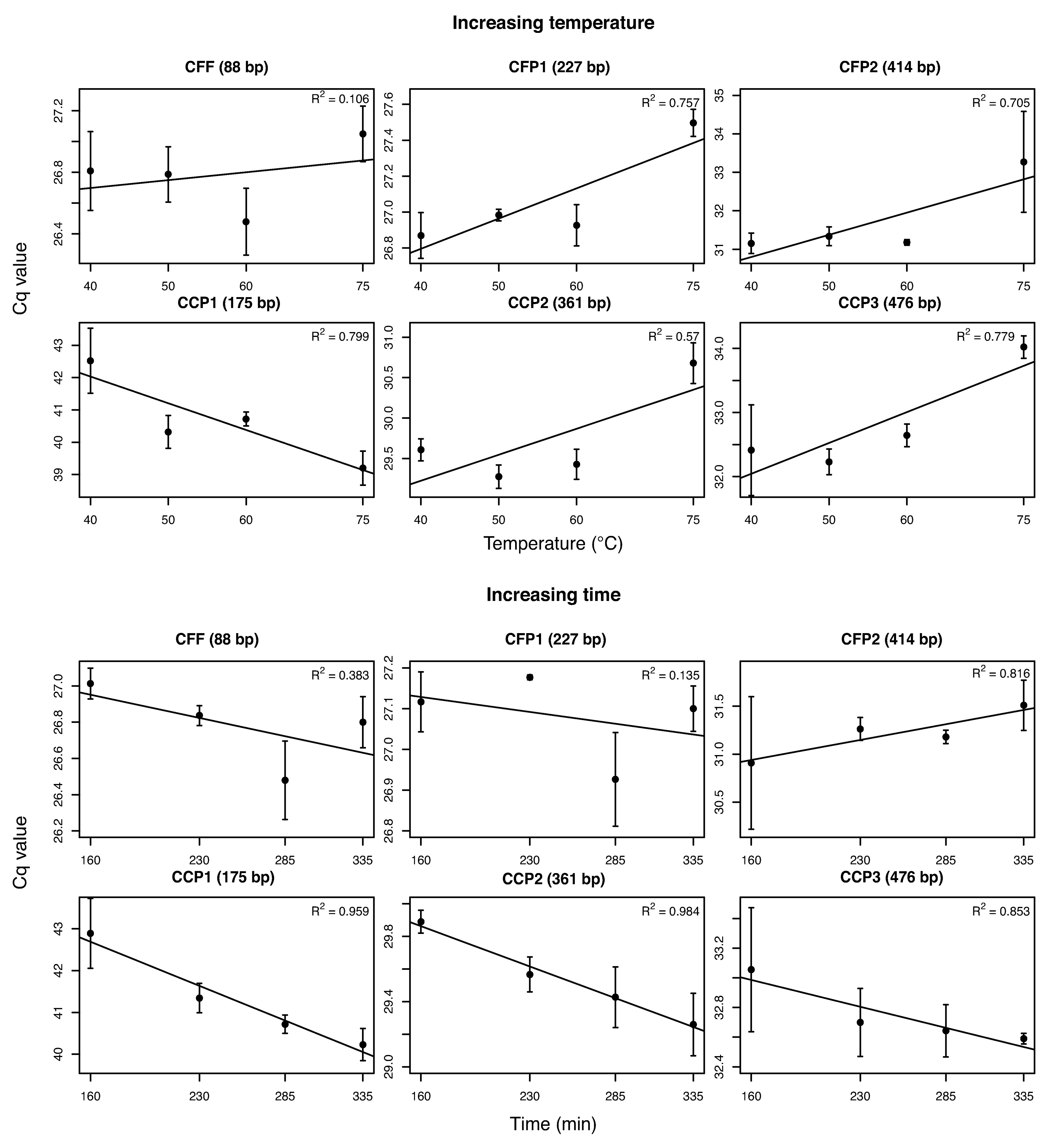

Supplement: S3 Fig — Upper panel: protocol changed in conversion temperature (S7 Table). Lower panel: protocol changed in conversion time (S8 Table). Results are given as Cq values ± SD. (TIF) [file pone.0199091.s013.tif]

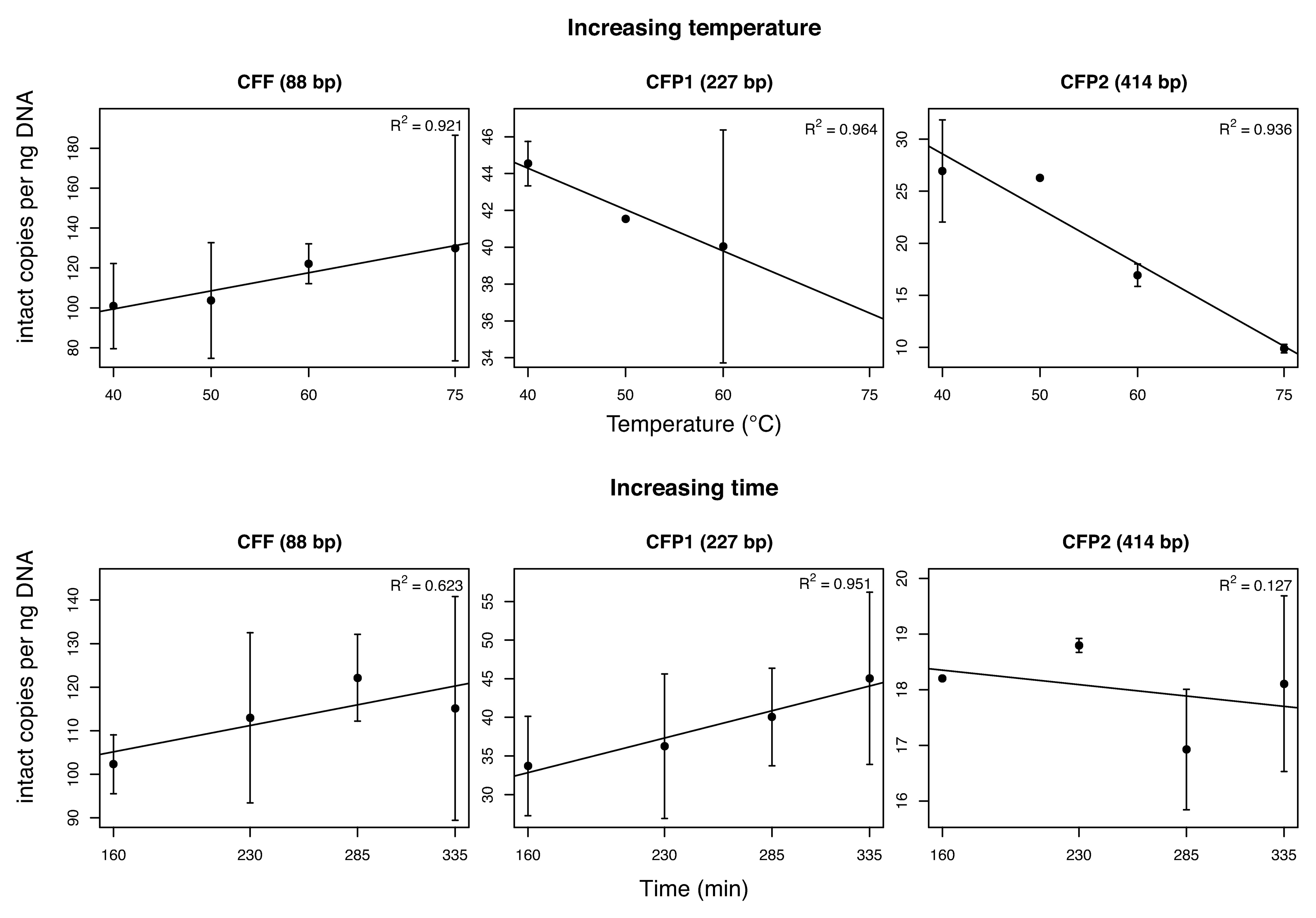

Supplement: S4 Fig — Upper panel: protocol changed in conversion temperature (S7 Table). Lower panel: protocol changed in conversion time (S8 Table). Results are given as number of intact copies per ng bisulfite treated DNA measured by dPCR ± SD. (TIF) [file pone.0199091.s014.tif]

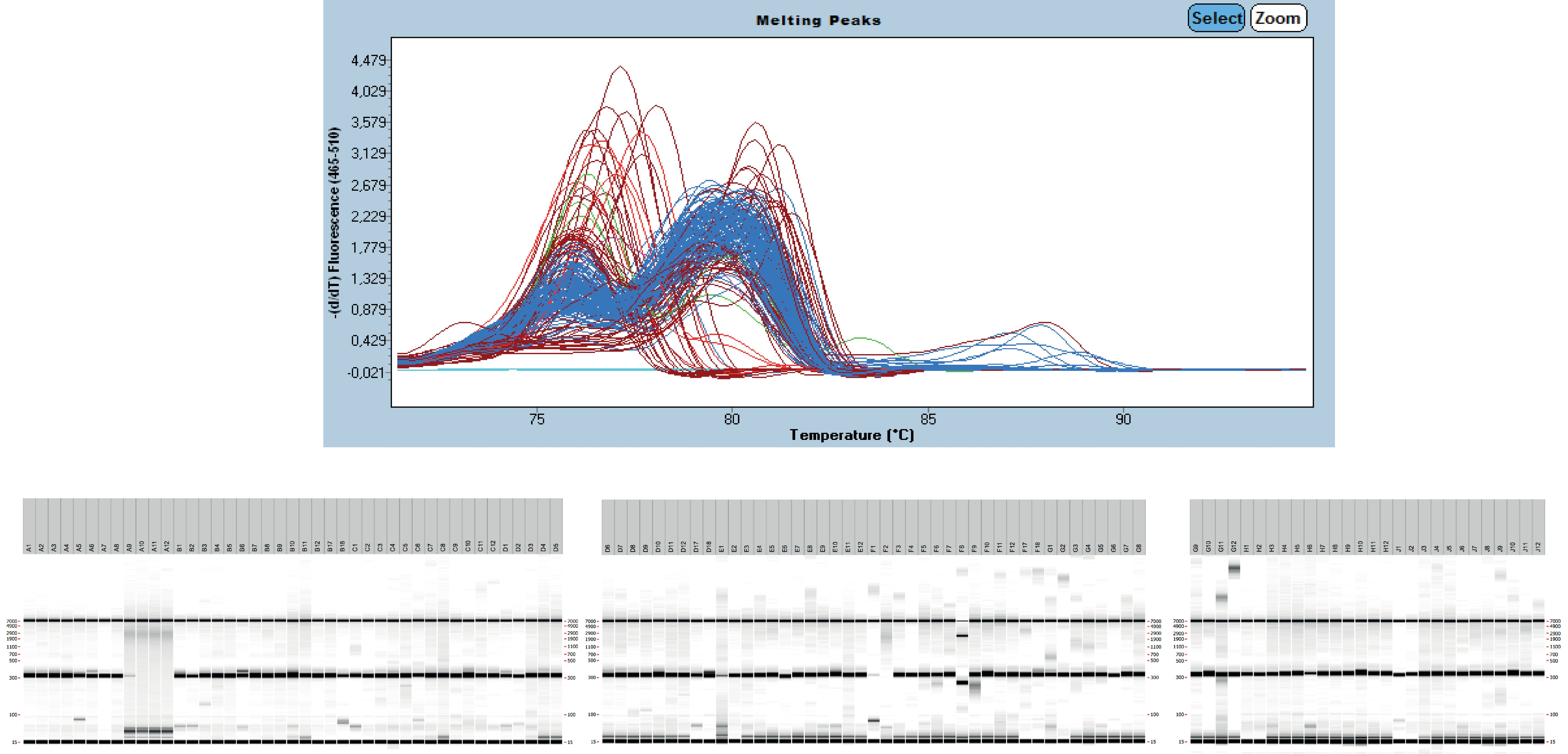

Supplement: S5 Fig — Example of the melting curves (upper panel) and the Caliper LabChip GX results (lower panel) after qPCR showing aspecific melting curves, but specific bands for the same reaction: CCP1_after. (TIF) [file pone.0199091.s015.tif]

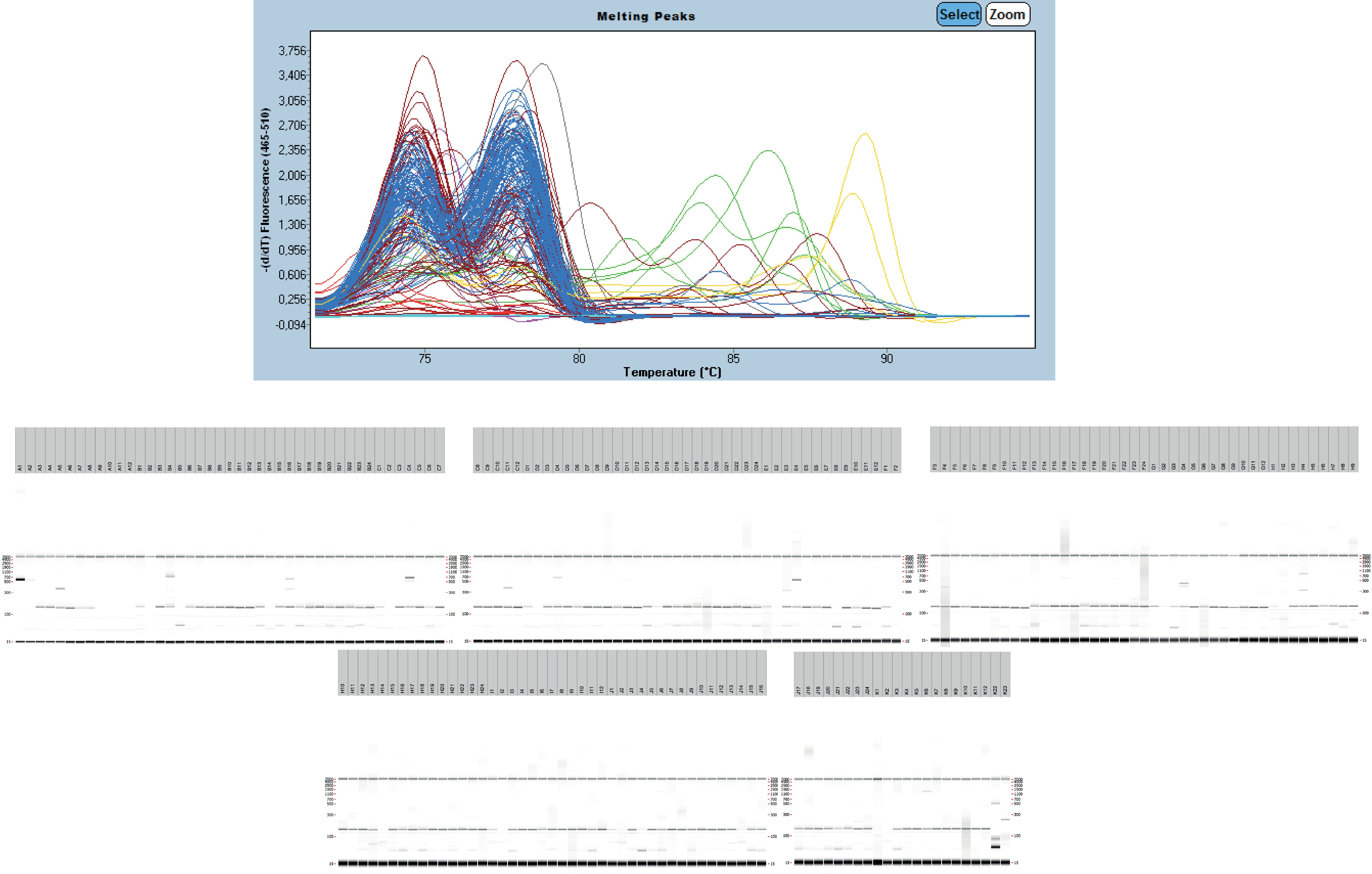

Supplement: S6 Fig — Example of the melting curves (upper panel) and the Caliper LabChip GX results (lower panel) after qPCR showing aspecific melting curves, but specific bands for the same reaction: CCP2_after. (TIF) [file pone.0199091.s016.tif]
